# Diversity of the Hydroxylamine Oxidoreductase (HAO) Gene and Its Enzyme Active Site in Agricultural Field Soils

**DOI:** 10.1264/jsme2.ME23068

**Published:** 2023-12-13

**Authors:** Tsubasa Ohbayashi, Yong Wang, Luciano Nobuhiro Aoyagi, Shintaro Hara, Kanako Tago, Masahito Hayatsu

**Affiliations:** 1 Institute for Agro-Environmental Sciences, National Agriculture and Food Research Organization (NARO), 305–8604, Tsukuba, Japan

**Keywords:** ammonia-oxidizing bacteria, comammox, nitrification, HAO

## Abstract

Nitrification is a key process in the biogeochemical nitrogen cycle and a major emission source of the greenhouse gas nitrous oxide (N_2_O). The periplasmic enzyme hydroxylamine oxidoreductase (HAO) is involved in the oxidation of hydroxylamine to nitric oxide in the second step of nitrification, producing N_2_O as a byproduct. Its three-dimensional structure demonstrates that slight differences in HAO active site residues have inhibitor effects. Therefore, a more detailed understanding of the diversity of HAO active site residues in soil microorganisms is important for the development of novel nitrification inhibitors using structure-guided drug design. However, this has not yet been examined. In the present study, we investigated *hao* gene diversity in beta-proteobacterial ammonia-oxidizing bacteria (β-AOB) and complete ammonia-oxidizing (comammox; *Nitrospira* spp.) bacteria in agricultural fields using a clone library ana­lysis. A total of 1,949 *hao* gene sequences revealed that *hao* gene diversity in β-AOB and comammox bacteria was affected by the fertilizer treatment and field type, respectively. Moreover, *hao* sequences showed the almost complete conservation of the six HAO active site residues in both β-AOB and comammox bacteria. The diversity of nitrifying bacteria showed similarity between *hao* and *amoA* genes. The *nxrB* amplicon sequence revealed the dominance of *Nitrospira* cluster II in tea field soils. The present study is the first to reveal *hao* gene diversity in agricultural soils, which will accelerate the efficient screening of HAO inhibitors and evaluations of their suppressive effects on nitrification in agricultural soils.

Nitrification is a key process in the biogeochemical nitrogen cycle, wherein ammonia is aerobically oxidized to nitrate. In agricultural soils, this process is driven by four different microbial groups: ammonia-oxidizing archaea (AOA), ammonia-oxidizing bacteria (AOB), nitrite-oxidizing bacteria (NOB), and complete ammonia-oxidizing (comammox) bacteria (Hayatsu *et al.*, 2021). These nitrifying microorganisms are closely associated with the application of nitrogen fertilizers because the chemoautotrophic bacteria proliferate by obtaining energy from the oxidization of ammonia and/or nitrite (Norton and Ouyang, 2019). Nitrification activity increases in response to nitrogen fertilization amendments, and often leads to environmental pollution via nitrate leaching as well as nitrogen loss from agricultural fields. Nitrogen use efficiency in crops is less than 50% of nitrogen fertilizer input due to nitrogen loss, including nitrification (Zhang *et al.*, 2021).

Nitrification and denitrification in the nitrogen cycle are major emission pathways for the greenhouse gas N_2_O, and these pathways are highly dependent on environmental factors, such as the substrate type and its availability, soil oxygen concentrations, and water contents in soil (Khalil *et al.*, 2004). Previous studies reported the existence of agricultural soils with high N_2_O emission derived from nitrification or denitrification pathways. For example, N_2_O emission via the nitrification pathway is dominant in soils with a water-filled pore space (WFPS) less than 60% (Bollmann and Conrad, 1998; Bateman and Baggs, 2005; Baggs *et al.*, 2010; Huang *et al.*, 2014). It has also been suggested to occur in lower pH soils (Liu *et al.*, 2016). On the other hand, elevations in denitrification activity and denitrification-derived N_2_O emissions have been reported when soil moisture increases due to rainfall and O_2_ concentrations decrease (Butterbach-Bahl *et al.*, 2013). High organic matter also increases N_2_O emissions derived from denitrification in soils (Thomson *et al.*, 2012).

AOB generate N_2_O through two metabolic pathways, hydroxylamine oxidization and nitrifier denitrification (Ward *et al.*, 2011). AOB play major roles in the nitrification of agricultural soils treated with inorganic nitrogen fertilizers and are responsible for N_2_O emissions (Prosser *et al.*, 2020). In contrast, the N_2_O emission potential of comammox bacteria is considered to be very low based on *in vitro* pure culture experiments (Kits *et al.*, 2019). Similar findings were confirmed in soil microcosm experiments (Tan *et al.*, 2022). However, limited information is currently available on the N_2_O emission potential of comammox bacteria. Therefore, further studies are needed to demonstrate the contribution of N_2_O emissions by comammox bacteria.

Several nitrification control methods, such as polymer-coated fertilizers (PCF) and nitrification inhibitors (NIs), have been proposed to reduce N_2_O emissions. PCF reduce nitrification activity by controlling the release of ammonium into the soil via a physical barrier and consequently reduce N_2_O emissions. NIs decelerate nitrification activity for nitrogen from fertilizers applied to soil by temporarily inhibiting ammonia oxidation. NIs are considered to be the most effective N_2_O mitigation method, and three commercially available NIs are now widely used: 2-chloro-6-(trichloromethyl) pyridine (Nitrapyrin), dicyandiamide (DCD), and 3,4-dimethylpyrazole phosphate (DMPP). However, the reduction of N_2_O emissions by these NIs was previously reported to range between 31% and 44% (Akiyama *et al.*, 2010) and 46% and 53% (Fan *et al.*, 2022). Therefore, the development of more effective NIs is urgently desired.

In nitrification, ammonia oxidation occurs in multiple steps catalyzed by ammonia monooxygenase (AMO) and hydroxylamine oxidoreductase (HAO). AMO catalyzes the oxidation of NH_3_ to NH_2_OH, while HAO catalyzes the oxidation of NH_2_OH to NO. The enzyme responsible for the oxidation of NO to NO_2_^–^ has not yet been identified (Ward *et al.*, 2011; Caranto and Lancaster, 2017). The majority of commercially available NIs target AMO. However, difficulties are associated with the purification and crystallization of AMO, and the three-dimensional protein structure of this enzyme is unknown (Hayatsu *et al.*, 2021). In contrast, HAO is a periplasmic enzyme and its steric structure has been elucidated (Igarashi *et al.*, 1997; Cedervall *et al.*, 2013; Maalcke *et al.*, 2014; Nishigaya *et al.*, 2019); therefore, the development of novel and more effective inhibitors is possible using structure-guided drug design.

A previous study revealed the three-dimensional protein structure of HAO in the gamma-proteobacterial AOB (γ-AOB) *Nitrosococcus oceani*, possessing six active site residues, similar to HAO in the beta-proteobacterial AOB (β-AOB) *Nitrosomonas europaea* (Nishigaya *et al.*, 2019). However, since two of the six HAO active site residues are different between β-AOB and γ-AOB, the effects of the HAO inhibitor phenylhydrazine differ: 40% inhibition against β-AOB HAO and almost no inhibition against γ-AOB HAO‍ ‍(Nishigaya *et al.*, 2016, 2019). The three-dimensional protein structure of comammox-HAO currently remains unclear. A genome ana­lysis revealed that comammox bacteria have the β-AOB type of HAO (Palomo *et al.*, 2018). Therefore, a more detailed understanding of the diversity of HAO active site residues in soil microorganisms involved in nitrification is important for the development of HAO inhibitors. However, *hao* gene diversity in agricultural soil has not yet been investigated.

To reveal *hao* gene diversity in agricultural soil, the abundance and community structure of AOB and comammox bacteria possessing HAO need to be examined. The abundance and diversity of AOB and AOA are generally investigated using the 16S rRNA gene and *amoA* encoding the alpha-subunit of ammonia monooxygenase as a phylogenetic marker gene (Purkhold *et al.*, 2000; Hatzenpichler, 2012; Monteiro *et al.*, 2014; Aigle *et al.*, 2019). The 16S rRNA gene and *nxrB* encoding the beta-subunit of nitrite oxidoreductase are typically used for NOB and comammox bacteria (Pester *et al.*, 2014).

In the present study, we designed PCR primer sets for *hao* genes containing HAO active sites and then investigated *hao* gene diversity in soils from agricultural fields in which different crops were cultivated under distinct nitrogen fertilization regimes. The *hao* gene sequences obtained in the present study provide insights into the diversity of HAO active sites in β-AOB and comammox bacteria. Furthermore, the community structure and abundance of AOB and comammox bacteria in soil samples were analyzed by amplicon sequencing and quantitative PCR (qPCR) using the *amoA* gene for AOB and comammox bacteria, and the *nxrB* gene for canonical-NOB and comammox bacteria. We also investigated the relationship between the AOB and comammox bacterial community structure and HAO diversity in soils under different nitrogen fertilization regimes.

## Materials and Methods

### Soil samples

Three agricultural fields (cabbage, soybean, and tea) were selected for this study. The cabbage field was located at the Institute of Fruit Tree and Tea Science, National Agriculture and Food Research Organization (NARO), Tsukuba, Ibaraki, Japan (36°02′N, 140°11′E). The soybean field was located at the Institute for Agro-Environmental Sciences, NARO Tsukuba, Ibaraki, Japan (36°01′N, 140°07′E). The tea field was located at Kagoshima Prefectural Institute For Agricultural Development, Minamisatsuma, Kagoshima, Japan (31°48′N, 130°34′E). The soil in each agricultural field was treated with chemical or organic nitrogen fertilizer. Details on fertilizer application are shown in Table S1. The soybean field to which the organic fertilizer was applied had been under non-tillage management for thirty years. The soil type in all three agricultural fields was Andosol. Surface soil at a depth of 0–1‍ ‍cm was removed. Using a small sterile shovel, soil samples were collected at a depth of 1–10‍ ‍cm in triplicate and sieved through a mesh with a pore size of 2‍ ‍mm. All soil samples were stored at –‍80°C until DNA extraction or at 4°C for an ammonia oxidation potential ana­lysis within 7 days. Air-dried soil was used to analyze soil properties.

### Characterization of soil properties

Soil properties were analyzed as previously described (Tago *et al.*, 2015). Briefly, soil pH was assessed in 1:2.5 (w/v) soil/water suspensions. Total carbon and nitrogen contents were measured using an elemental analyzer (2400II CHNS/O, PerkinElmer). Available phosphate was extracted from soil suspensions to 2‍ ‍mM sulfuric acid (w/v) for 30‍ ‍min and the concentration was calorimetrically determined using the molybdenum blue method. To measure the contents of ammonium nitrogen and nitrate nitrogen, a 10-g soil sample was extracted with 100‍ ‍mL of 2 M KCl, and the soil suspension was filtered through Whatman paper No. 10 filter paper. Ammonium nitrogen was measured by a continuous flow analyzer (TRRACS, Bran+Luebbe) using the indophenol blue method. Nitrate nitrogen was analyzed using the copper cadmium reduction method.

### Ammonia oxidation potential ana­lysis

Ammonia oxidation potential was measured based on the nitrite accumulation rate in the presence of an inhibitor of nitrite oxidization, potassium chlorate (Belser and Mays, 1980). Briefly, 2.5‍ ‍g soil was suspended in 10‍ ‍mL of 1‍ ‍mM phosphate buffer (pH 7.0) containing 1‍ ‍mM (NH_4_)_2_SO_4_ and 10‍ ‍mM KClO_3_ and incubated at 25°C with continuous rotation at 150‍ ‍rpm for several hours. A 1-‍mL soil suspension incubated for several hours was centrifuged at 10,000×*g* at 4°C for 10‍ ‍min. The concentration of accumulated NO_2_^–^ in the supernatant was spectrophotometrically assessed using the Griess-Ilosvay method. Nitrification rates were calculated based on the accumulation of nitrite (nmole h^–1^ [g dry soil]^–1^) (Keeney and Nelson, 1982).

### DNA extraction from soil samples

Soil DNA was extracted from 0.4‍ ‍g soil with the Fast DNA SPIN Kit for Soil (Qbiogene) as described in previous studies (Takada-Hoshino and Matsumoto, 2004; Morimoto *et al.*, 2011). The extracted DNA sample was purified by MicroSpin S-400 HR columns (GE Healthcare) and the DNA Clean and Concentrator-25 kit (Zymo Research).

### Quantitative PCR (qPCR)

Extracted DNA was subjected to qPCR using SYBR Premix Ex Taq (Takara Bio) and 200 nM of primers. The primers and PCR temperature profile used for AOA-*amoA* and AOB-*amoA* genes were described in previous studies (Rotthauwe *et al.*, 1997; Nicolaisen and Ramsing, 2002; Tourna *et al.*, 2008; Morimoto *et al.*, 2011; Yang *et al.*, 2017; Table S2). qPCR of the comammox-*amoA* and *nxrB* genes was conducted using two primer sets: comaA-*amoA*-F (5′–CBKCNTGGTGGTGGTGGTC–3′) and comaA-*amoA*-R (5′–AGCCCATRTAGTCNGCCC–3′), and *nxrB*-F (5′–GTGGTGGAACAAYGTSGARAC–3′) and *nxrB*-R (5′–GCATCGABGTNGSVGTRTC–3′), respectively. The PCR temperature profile was set to 95°C for 2‍ ‍min, followed by 40 cycles of 95°C for 30‍ ‍s, 65°C in comammox-*amoA* and 60°C in *nxrB* for 30‍ ‍s, and 72°C for 1‍ ‍min using the StepOnePlus Real-Time PCR System (Applied Biosystems). The copy numbers of the three target genes were calculated based on a standard curve generated using a dilution series of linearized pGEM-T Easy plasmids (Promega) containing clones of each PCR fragment.

### Clone library ana­lysis of β-AOB and comammox *hao* genes

The PCR primers covering the β-AOB and comammox HAO enzyme active sites were designed as follows: Hao-pira-F (5′–TGCCAYACCAACCAGAACAA–3′) and *hao*-pira-R (5′–ATCTTGGTGTTYTCGTCCATG–3′), and *hao*-AS-F2 (5′–TGCCAYRYCMABCARAAYAAG–3′) and *hao*-AS-R2 (5′–TCMTCRTCCATGATTTCSACA–3′), respectively (Fig. S1). The 770-bp and 758-bp fragments of the β-AOB and comammox *hao* genes, respectively, were amplified using soil DNA samples, the aforementioned primer sets (Table S2), and TaKaRa Ex Taq (Takara Bio). The PCR‍ ‍temperature profile was set to 94°C for 2‍ ‍min, followed by 30‍ ‍cycles at 94°C for 30‍ ‍s, 50°C in β-AOB-*hao* or 55°C in comammox-*hao* for 30‍ ‍s, and 72°C for 1‍ ‍min using the Applied Biosystems Veriti thermal cycler (Applied Biosystems). PCR products were purified using a QIAquick PCR Purification Kit (Qiagen), ligated into pGEM-T Easy Vector Systems (Promega), and cloned into *Escherichia coli* JM109 competent cells (Takara Bio). Cloned PCR products were sequenced at the Takara Bio Biomedical Center (Takara Bio).

### Amplicon sequencing

Eighteen soil DNA samples (6 soil samples in triplicate) were subjected to PCR amplification of the AOB-*amoA*, comammox-*amoA*, and *nxrB* genes for an amplicon sequence ana­lysis. PCR amplification was performed using TaKaRa Ex Taq (Takara Bio) and fusion primers containing adaptor sequences, keys, multiplex identifiers, and gene-specific sequences (Table S2). The PCR temperature profile was set to initial denaturation at 94°C for 2‍ ‍min, followed by 25 cycles at 94°C for 30‍ ‍s, the annealing temperature of each gene (Table S2) for 30‍ ‍s, and a final extension at 72°C for 1‍ ‍min using the Applied Biosystems Veriti thermal cycler (Applied Biosystems). PCR products were purified using the QIAquick PCR purification kit (Qiagen), and gel extraction was performed with the QIAquick Gel Extraction kit (Qiagen). The quality and quantity of the purified PCR amplicons were analyzed using an Agilent 2100 Bioanalyzer (Agilent Technologies) and the Quant-iT PicoGreen dsDNA Assay kit (Thermo Fisher Scientific), respectively. The amplicon sequence was analyzed by the MiSeq sequencer using Miseq Reagent kit v2 (Illumina), according to the manufacturer’s instructions.

### Sequence ana­lysis and phylogenetic assignment

The sequences of the β-AOB and comammox *hao* genes from the clone library ana­lysis were assigned to operational taxonomical units (OTU) based on a 95% sequence identity threshold and >1% in all sequences using the cd-hit-est program on the server (Huang *et al.*, 2010). The most similar bacterial species in OTUs were identified by a BLASTX search of the NCBI database. The representative nucleotide sequences of each OTU were used for a phylogenetic ana­lysis. Amplicon sequence quality was examined using the DADA2 package (Callahan *et al.*, 2016) in R software (R Core Team, 2021). After the removal of low quality and chimeric sequences, non-target sequences were identified by a BLAST search against a target gene at the FunGene database and removed using Mothur (Schloss *et al.*, 2009). Filtered sequences were then clustered using Mothur into OTUs with cut-off values of 0.05 for AOB-*amoA*, 0.06 for comammox-*amoA*, and 0.04 for *nxrB* genes. The representative nucleotide sequences in each OTU were aligned with the MAFFT program on the EMBL-EBI server (Li *et al.*, 2015). Phylogenetic trees were generated for the *amoA*, *hao*, and *nxrB* genes using the maximum likelihood (ML) method with the removal of gap-including and ambiguous sites and with a bootstrap ana­lysis (1,000 replicates) in MEGA software version 10.1.8 (Kumar *et al.*, 2018; Stecher *et al.*, 2020). We selected the Tamura-Nei model of nucleotide substitutions with gamma distributed and invariant sites (G+I) (Tamura and Nei, 1993).

### Confirmation of HAO enzyme active sites

The 1,112 β-AOB-*hao* sequences derived from the clone library ana­lysis and *Nitrosospira multiformis* ATCC25196 [CP000103] as a reference gene were aligned using the MAFFT program and translated into amino acid sequences using MEGA version 10.1.8. The six residues at the HAO active sites in the aligned sequences were manually confirmed using Jalview software ver. 2.11.2.4 (Waterhouse *et al.*, 2009). A total of 837 comammox-*hao* sequences and *Nitrospira inopinata* ENR4 [LN885086] were aligned, and their HAO active site residues were verified as described above.

### Statistical ana­lysis

Correlations between qPCR data and soil parameters were estimated by calculating Spearman’s ρ values using R software.

### Nucleotide sequence accession numbers

The nucleotide sequences of the *hao* genes obtained in the present study were deposited in the DDBJ/Genbank/EBI databases under the accession numbers LC724068–LC726016. The amplicon sequence reads reported in this study have been deposited in the DDBJ Sequence Read Archive (DRA) under the accession number: DRA014735 (See also Table S1).

## Results

### Soil physicochemical properties and ammonia oxidation potential in soil samples

Tea field soils had lower pH and higher total carbon, total nitrogen, and available phosphate than cabbage and soybean field soils (Table 1). The type of nitrogen fertilizer affected several soil properties. Organic fertilizer treatment increased NO_3_-N concentrations in the three agricultural fields. Total carbon and total nitrogen contents were higher in cabbage and soybean field soils treated with organic fertilizer. The content of NH_4_-N was higher in soybean and tea field soils treated with organic fertilizer (Table 1). In all fields, ammonia oxidation potential (AOP) levels were two- to six-fold higher with the organic fertilizer treatment than with the chemical fertilizer treatment (Table 1).

### Abundance of soil microbes involved in nitrification

The microbial population involved in nitrification in each agricultural field was investigated by qPCR. The abundances of AOA-*amoA* and AOB-*amoA* were similar, ranging from 9.7×10^6^ to 3.9×10^7^ and from 3.7×10^5^ to 5.4×10^6^ gene copies (g‍ ‍dry‍ ‍soil)^–1^, respectively, except for the tea field treated with chemical fertilizer, which showed the lowest abundances of 1.7×10^4^ and 1.8×10^5^ gene copies, respectively (Table 2). On the other hand, comammox-*amoA* in all three fields ranged from 1.5×10^7^ to 1.3×10^8^ gene copies (g‍ ‍dry‍ ‍soil)^–1^, *i.e.*, markedly higher than AOA-*amoA* and AOB-*amoA*, except for chemical fertilizer-treated cabbage soil (9.1×10^5^ gene copies). All three field soils had similar values ranging from 3.9×10^6^ to 2.7×10^7^ *nxrB* gene copies (g‍ ‍dry‍ ‍soil)^–1^ involved in nitrite oxidation (Table 2).

### Relationship between soil properties and abundance of nitrifying microorganisms

We examined the relationships between eight parameters of soil properties and the abundance of four microbial gene copies in qPCR data (Table S3). AOP positively correlated with the abundances of AOB-*amoA* and *nxrB*, but not with the abundances of AOA-*amoA* or comammox-*amoA* (Table S3). In other soil parameters, the abundance of AOA-*amoA* positively correlated with pH, but negatively correlated with total nitrogen, available phosphate, and water content. The abundance of comammox-*amoA* positively correlated with total carbon, total nitrogen, nitrate-nitrogen, and water content (Table S3). The abundance of *nxrB* positively correlated with pH (Table S3).

### The *hao* gene diversity of β-AOB and comammox bacteria

To investigate the *hao* gene diversity of β-AOB and comammox bacteria in soil samples collected from cabbage, soybean, and tea fields, we designed two primer sets against the β-AOB and comammox *hao* genes, respectively, including six amino acid residues of the HAO enzyme active site (Fig. S1), and then conducted a clone library ana­lysis of six soil samples. We obtained 1,949 *hao* sequences, derived from 1,112 and 837 clones in β-AOB and comammox, respectively (Tables S4 and S5). The 1,949 assembled sequences were assigned to 30 OTUs based on the 95% sequence identity threshold and >1% detection of all sequences. Sixteen β-AOB-*hao* OTUs were identified as *Nitrosospira* spp., whereas 14 comammox-*hao* OTUs were members of *Nitrospira* in the NCBI database using a BLASTX search.

**Fig. 1.**
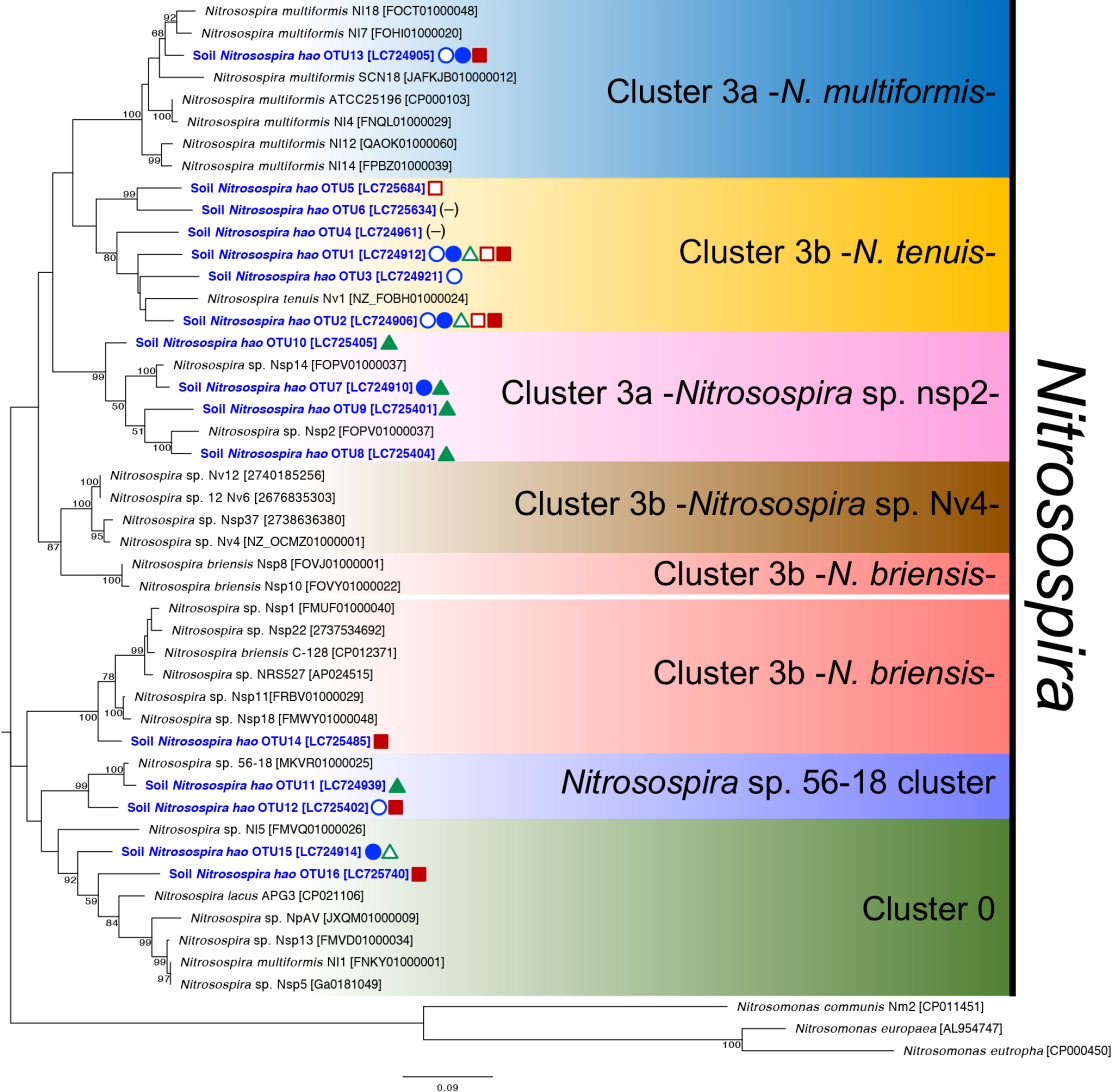
Molecular phylogenetic ana­lysis of β-AOB-*hao* gene diversity in agricultural field soils. A maximum-likelihood tree was generated based on 719 aligned nucleotide sites of the *hao* gene. Maximum-likelihood bootstrap values (%) were calculated with 1,000 replicates, and bootstrap values >50 are shown at the tree nodes. We used reference sequences reported in a previous study (Aigle *et al.*, 2019). Accession numbers in the DNA database (DDBJ/EMBL/GenBank or JGI) are shown in square brackets. Representative β-AOB-*hao* OTUs are shown in blue with a bold case font. Symbols indicate >5% relative abundance in the six soil samples; open circle: cabbage field soil treated with chemical fertilizer; closed circle: cabbage field soil treated with organic fertilizer; open triangle: soybean field soil treated with chemical fertilizer; closed triangle: soybean field soil treated with organic fertilizer; open square: tea field soil treated with chemical fertilizer; closed square: tea field soil treated with organic fertilizer; dash: none of any field. Symbol colors indicate each field: blue, cabbage field; green, soybean field; red, tea field.

**Fig. 2.**
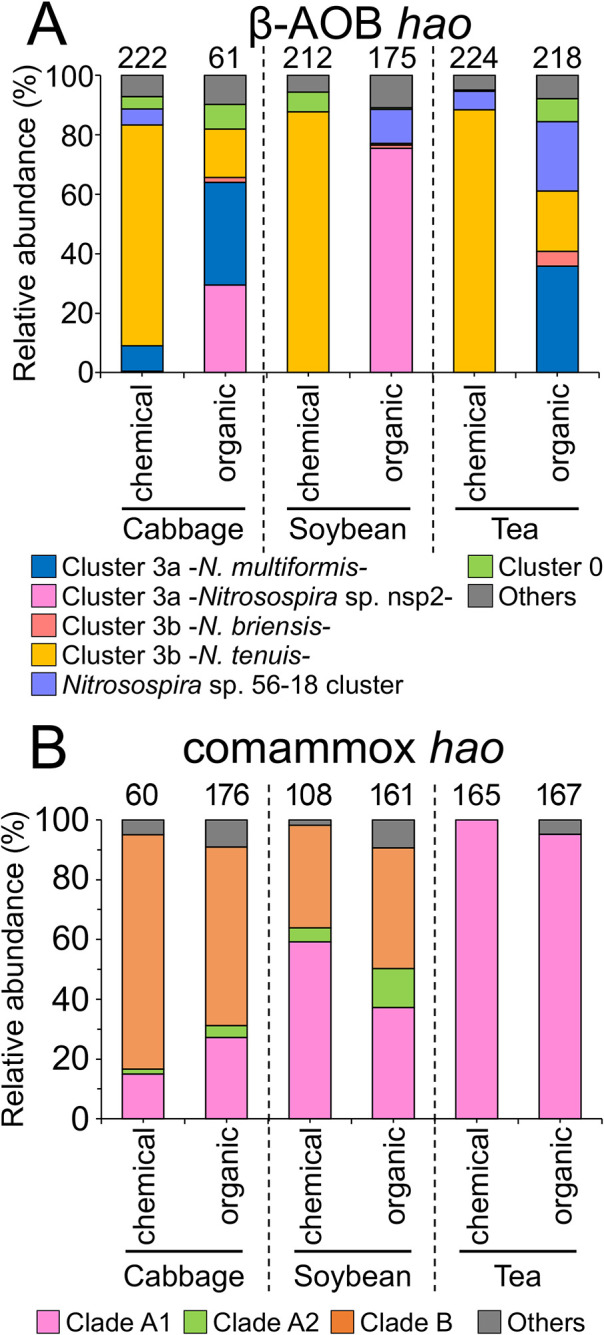
Relative abundance of *hao* gene diversity in agricultural fields in (A) β-AOB and (B) comammox bacteria. Subclades were identified based on each phylogenetic tree (Fig. 1 and 3). The total number of clones in each soil is shown in the graphs, and details are provided in Table S4 and Table S5.

We performed a mole­cular phylogenetic ana­lysis of the 16 β-AOB-*hao* OTUs and other reference sequences in the β-AOB-*hao* gene. The 16 OTUs were classified into six subgroups in the genus *Nitrosospira* (Fig. 1). Since cluster 3b was more diverse than the other clusters, it was divided into three subgroups: cluster 3b -*N. tenuis-*, cluster 3b -*N. briensis-*, and cluster 3b -*Nitrosospira* sp. Nv4-. The relative abundance of the AOB-*hao* gene in the six soil samples at the AOB subgroup level is shown in Fig. 2A. It revealed that the β-AOB-*hao* gene diversity was altered more markedly by fertilizer type than field type. *Nitrosospira* cluster 3b -*N. tenuis*- was dominant in all three chemical fertilizer-treated soils, but was present at a low level in organic fertilizer-treated cabbage and tea field soils (Fig. 2A). In the soybean field treated with organic fertilizer, *Nitrosospira* cluster 3a -*Nitrosospira* sp. nsp2- accounted for more than 70% of the population, and was also detected in the cabbage field treated with organic fertilizer. *Nitrosospira* sp. 56-18 cluster accounted for 25% of the population present in the tea field treated with organic fertilizer (Fig. 2A). Furthermore, 25% of the population present in cabbage and tea fields treated with organic fertilizer was *Nitrosospira* cluster 3a -*N. multiformis*- (Fig. 2A). *Nitrosospira* cluster 3b -*N. briensis*- and cluster 0 accounted for less than 10% in all soil samples (Fig. 2A). These results suggest that β-AOB-*hao* gene diversity in agricultural fields was markedly affected by the fertilizer treatment.

**Fig. 3.**
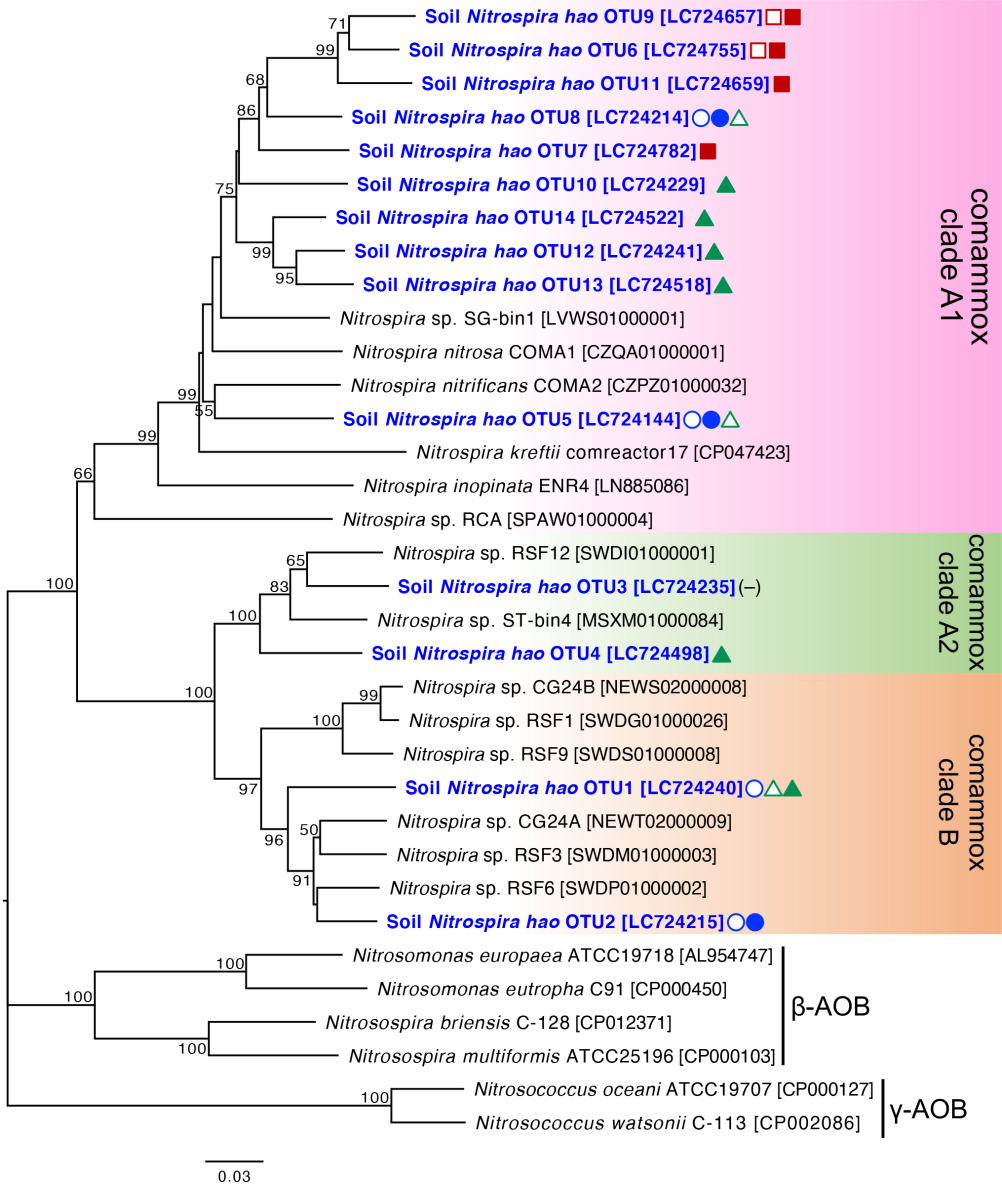
Molecular phylogenetic ana­lysis of comammox-*hao* gene diversity in agricultural field soils. A maximum-likelihood tree was generated based on 713 aligned nucleotide sites of the *hao* gene. Maximum-likelihood bootstrap values (%) were calculated with 1,000 replicates, and bootstrap values >50 are shown at the tree nodes. Accession numbers in the DNA database (DDBJ/EMBL/GenBank) are shown in square brackets. We referred to the nucleotide sequence information of comammox bacteria and its criteria reported in a previous study (Palomo *et al.*, 2022). Representative comammox *hao* OTUs are shown in blue with a bold case font. Symbols and colors are as shown in Fig. 1.

A mole­cular phylogenetic ana­lysis of the comammox-*hao* gene was performed based on 14 comammox-*hao* OTUs and other reference sequences in the comammox-*hao* gene. Fourteen OTUs were classified into three clusters in the genus *Nitrospira* (Fig. 3). Ten OTUs (OTU5–OTU14) belonged to comammox clade A1, two OTUs (OTU1 and OTU2) to comammox clade B, and two OTUs (OTU3 and OTU4) to comammox clade A2 (Fig. 3). The relative abundances of these comammox subgroups in agricultural fields are shown in Fig. 2B. In the four cabbage and soybean field soil samples, clade B accounted for 34%–79%, with small variations among soil samples (Fig. 2B). However, clade B was not detected in the tea field, in which clade A1 was dominant and accounted for more than 90% (Fig. 2B). Clade A2 was a minor population in all the field soil samples (Fig. 2B). Collectively, these results suggest that comammox-*hao* gene diversity was not significantly affected by the fertilizer treatment.

### Diversity of HAO enzyme active sites in β-AOB and comammox bacteria in agricultural fields

**Fig. 4.**
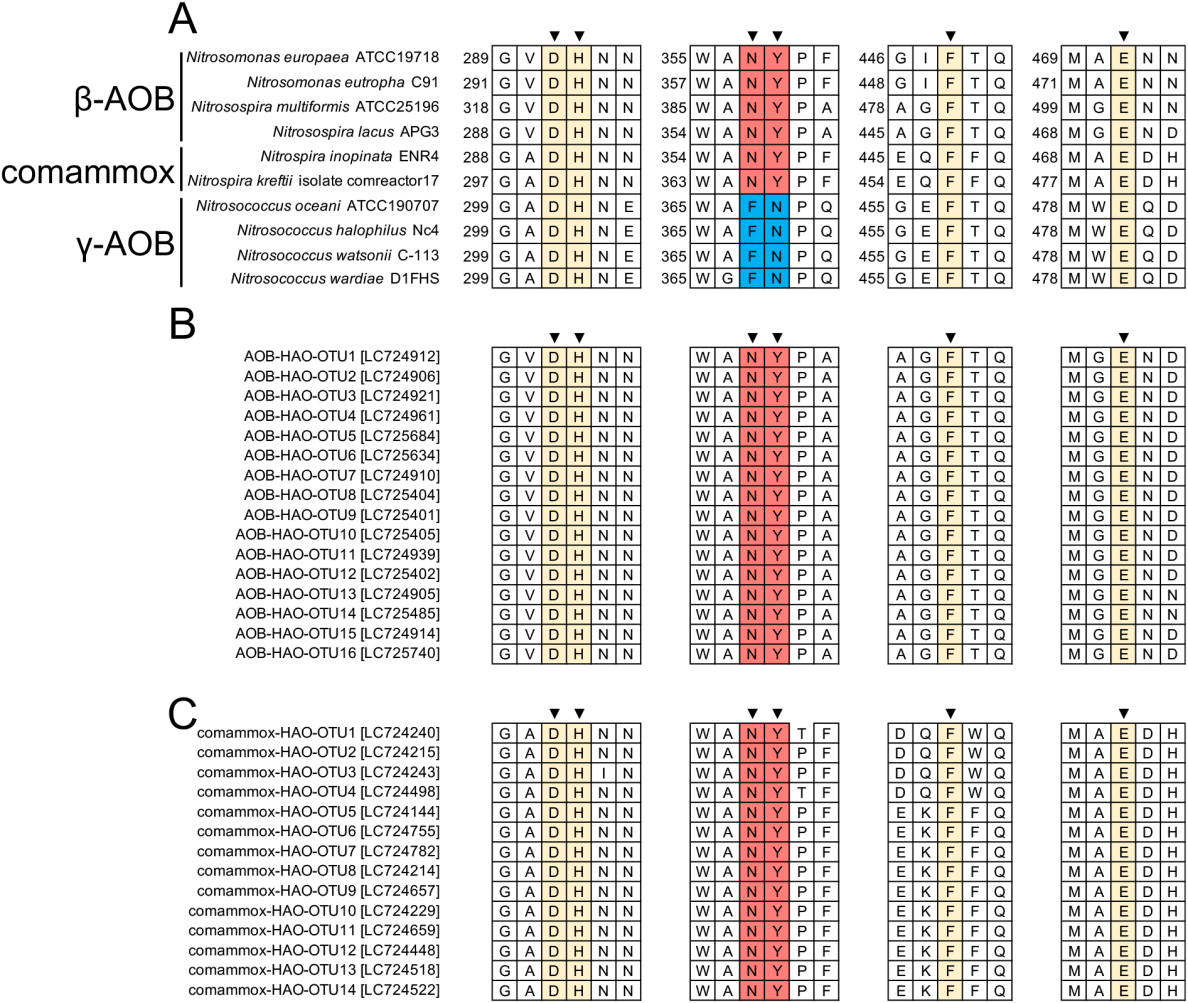
Confirmation of the hydroxylamine oxidoreductase (HAO) enzyme active site. (A) Multiple sequence alignment of HAO in (A) β-AOB, comammox, and γ-AOB, in (B) 17 β-AOB-*hao* OTUs, and in (C) 14 comammox-*hao* OTUs detected in agricultural field soils. Representatives of each OTU are shown. OTUs with different active site residues are summarized in Fig. S3. Colors indicate HAO active site residues conserved between β-AOB and comammox bacteria (red), with γ-AOB (blue), and among all three types of bacteria (yellow). Arrowheads indicate HAO active site residues. Accession numbers in the DNA database (DDBJ/EMBL/GenBank) are shown in square brackets.

Based on a protein crystal structure ana­lysis, the HAO enzyme in β-AOB possesses six residues at its active site (Nishigaya *et al.*, 2016; Fig. 4A). Although the protein crystal structure in comammox HAO has not yet been identified, its protein sequence identity strongly suggests that it possesses six active site residues, similar to β-AOB HAO (Fig. 4A). To confirm the diversity of the HAO enzyme active site in β-AOB and comammox bacteria in agricultural field soils, we confirmed the 1,949 *hao* sequences identified using a clone library ana­lysis. We found that 99.0% (1,101/1,112) of β-AOB-*hao* sequences corresponded to the six residues of the typical HAO active site in *N. europaea* (Fig. 4B). However, the remaining 1.0% (11/1,112) showed 1–2 residue substitutions at their HAO active sites (Fig. S3A). A diversity ana­lysis of the comammox-*hao* gene demonstrated that 99.3% (831/837) of the comammox-*hao* sequences had the same active site residues as those of the reference strain *Candidatus* Nitrospira inopinata (Fig. 4C), and only 0.7% (6/837) showed 1 residue substitution at the HAO active sites (Fig. S3B). Collectively, these results demonstrated that the six residues of the substrate-binding active sites in HAO were highly conserved in β-AOB and comammox bacteria, whereas HAO active site residues differed between β-AOB and γ-AOB.

### Amplicon sequence ana­lysis of the *amoA* gene in β-AOB and comammox bacteria

**Fig. 5.**
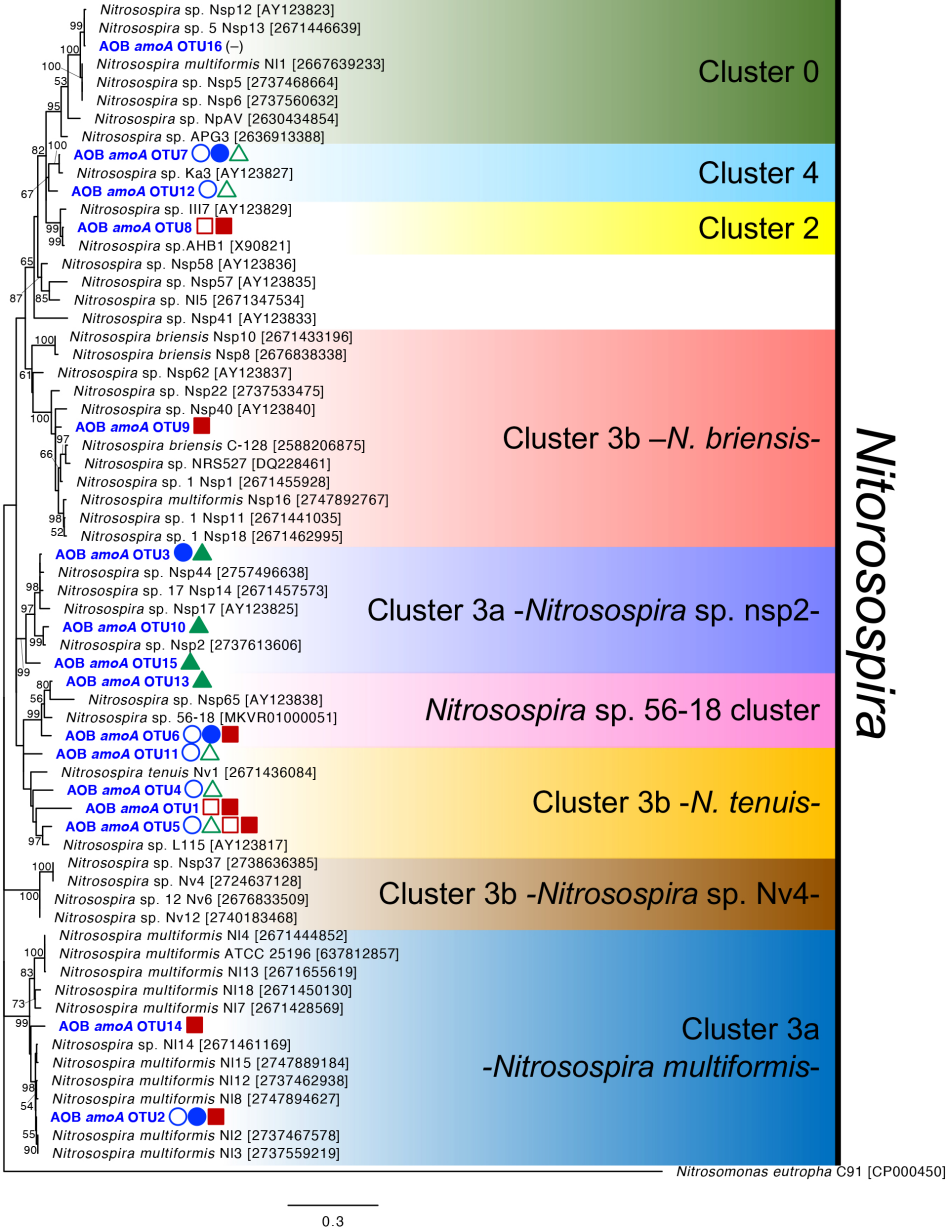
Molecular phylogenetic ana­lysis of AOB-*amoA* gene diversity in agricultural field soils. A maximum-likelihood tree was generated based on 452 aligned nucleotide sites of the *amoA* gene. Maximum-likelihood bootstrap values (%) were calculated with 1,000 replicates, and bootstrap values >50 are shown at the tree nodes. Accession numbers in the DNA database (DDBJ/EMBL/GenBank or JGI) are shown in square brackets. Representative AOB-*amoA* OTUs are shown in blue with a bold case font. Symbols and these colors are as shown in Fig. 1.

**Fig. 6.**
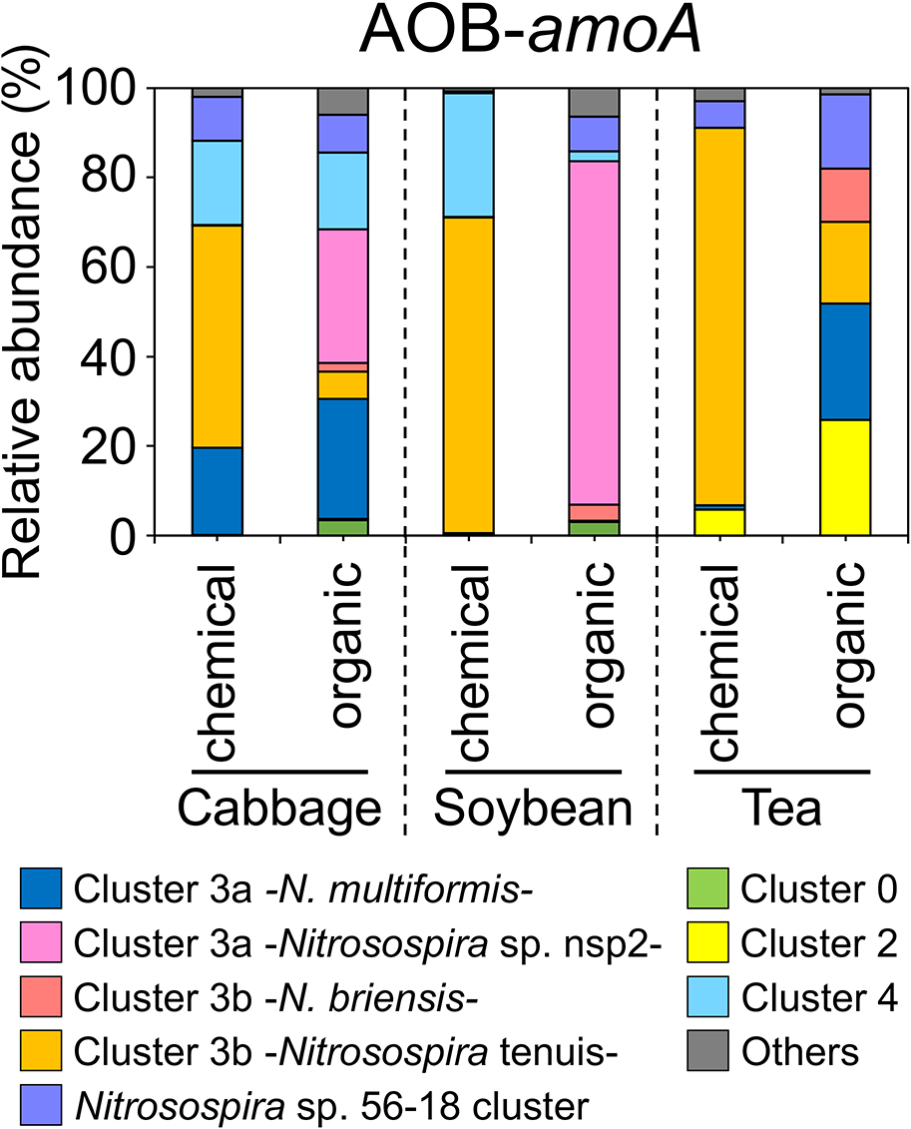
The relative abundance of AOB*-amoA* gene diversity in agricultural field soils. Subclades were identified based on phylogenetic divergence (Fig. 5). Detailed sequence reads detected in each cluster are shown in Table S6.

Phylogenetic ana­lyses of AOB have been performed using the marker gene, *amoA* (Purkhold *et al.*, 2000). Therefore, an *amoA* amplicon sequence ana­lysis was conducted to compare bacterial diversity between *amoA* and *hao* and confirm the diversity of nitrifying bacteria. High-quality AOB-*amoA* gene sequence reads were clustered into 16 OTUs based on a 95% sequence identity threshold and more than 1% coverage of the total reads (Table S6). Each soil sample contained 5–13 OTUs. Sixteen AOB-*amoA* OTUs were identified as members of *Nitrosospira* using a BLASTX search of the NCBI database. The nMDS plot showed that the AOB community structure was significantly affected by the field type and fertilizer treatment (Fig. S4). The AOB community in tea field soils markedly different from those in cabbage and soybean field soils, whereas the AOB community in cabbage and soybean fields was similar between chemical and organic fertilizer-treated soils (Fig. S4). We then performed a mole­cular phylogenetic ana­lysis of 16 AOB-*amoA* OTUs and other reference sequences in β-AOB-*amoA*. Within the genus *Nitrosospira*, 16 AOB-*amoA* OTUs were classified into eight subgroups (Fig. 5). The relative abundances of these *Nitrosospira* subgroups in the agricultural fields are shown in Fig. 6. The majority of AOB in cabbage, soybean, and tea fields treated with chemical fertilizer consisted of cluster 3b -*N. tenuis*-, whereas *Nitrosospira* cluster 3a -*Nitrosospira* sp. nsp2- was dominant in the soybean field treated with organic fertilizer. These results were consistent with the pattern revealed by the *hao* clone library ana­lysis. In cabbage and tea fields treated with organic fertilizer, no single cluster occupied more than 50% of the population, while the total of cluster 3a -*N. multiformis*- and cluster 3a -*Nitrosospira* sp. nsp2- was more than 50% in cabbage field soil treated with organic fertilizer, and the total of cluster 2 and cluster 3a -*N. multiformis*- was dominant in tea field soil treated with organic fertilizer (Fig. 6).

We also evaluated the diversity of comammox bacteria in soil samples using an *amoA* amplicon ana­lysis. High-quality comammox-*amoA* sequences clustered into 13 OTUs, and each soil sample contained 2–7 OTUs (Table S7). All of these bacteria were identified as members of *Nitrospira* spp. using a BLASTX search of the NCBI database. The nMDS ana­lysis demonstrated that comammox-*amoA* gene diversity was affected by the field soil rather than the fertilizer treatment (Fig. S5). A phylogenetic ana­lysis revealed that all 13 comammox-*amoA* OTUs belonged to comammox clade A; however, the node did not include well-known comammox strains including *N. inopinata* (Fig. S6). OTU2, OTU3, and OTU4 were dominant in cabbage field soil treated with chemical or organic fertilizers and soybean field soil treated with chemical fertilizer with minor variations in the OTU composition (Fig. S7). OTU6 occupied one fourth of the comammox population in cabbage field soil treated with organic fertilizer. The OTUs in soybean field soil treated with organic fertilizer were distinct from those in soybean field soil treated with chemical fertilizer. In the latter case, OTU8, OTU10, OTU11, and OTU12 were frequently detected (Fig. S7). On the other hand, OTU1 and OTU7 were dominant in tea field soil treated with chemical fertilizer. OTU1 accounted for more than 90%. OTU5 and OTU9 were detected in tea field soil treated with organic fertilizer (Fig. S7).

### Amplicon sequence ana­lysis of *nxrB* gene diversity in NOB

**Fig. 7.**
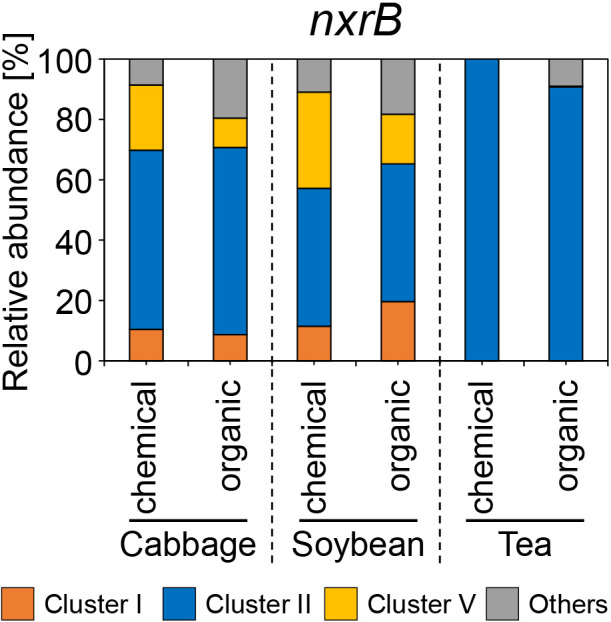
The relative abundance of *nxrB* gene diversity in agricultural field soils. Subclades were identified based on phylogenetic divergence (Fig. S10). Detailed sequence reads are shown in Table S8.

The genus *Nitrospira* was originally known as NOB and divided into six clusters. A part of *Nitrospira* cluster II is known as comammox bacteria (Daims *et al.*, 2015; van Kessel *et al.*, 2015). To confirm *Nitrospira* diversity, including canonical NOB and comammox bacteria, in soil samples, an amplicon sequence ana­lysis of the *nxrB* gene was conducted. The sequence reads of the six soil samples revealed 19 *nxrB* OTUs (Table S8). The *Nitrospira* community structure markedly differed between the tea field and cabbage and soybean fields (Fig. S8). In cabbage and soybean fields, the *Nitrospira* community structure of soybean field soil treated with organic fertilizer differed from that of the other three soils (Fig. S8A). The relative abundances of the 19 *nxrB* OTUs in each soil sample revealed that the tea field had a smaller number of OTUs than cabbage and soybean fields (Fig. S9). Four OTUs (OTU2, OTU8, OTU10, and OTU13) comprised *nxrB* in the tea field. Of these, OTU2, OTU10, and OTU13 were specifically detected in the tea field. OTU2 accounted for more than 80% of the *Nitrospira* population in the tea field soil treated with chemical fertilizer (Fig. S9). In contrast, the *nxrB* diversity was large in cabbage and soybean fields, detected in more than 10 OTUs per sample. Despite variations among samples, the majority (>50%) comprised five OTUs (OTU1, OTU3, OTU4, OTU5, and OTU6) in cabbage and soybean field soils (Fig. S9). The relative abundances of *Nitrospira* clusters in agricultural fields are shown in Fig. 7, which showed that *Nitrospira* cluster II was exclusive to tea field soils. On the other hand, clusters I and V, in addition to cluster II, were confirmed in cabbage and soybean field soils (Fig. 7).

## Discussion

The present study is the first to reveal *hao* gene diversity in β-AOB and comammox bacteria inhabiting various agricultural field soils. In addition, the *hao* gene sequences obtained herein showed the almost complete conservation of the six residues at the substrate-binding active site of the HAO enzyme in β-AOB and comammox bacteria (Fig. 4). The genus *Nitrospira*, originally known as NOB, is involved in nitrite oxidization. *Nitrospira* has been classified into six lineages, in which comammox bacteria were found to be a part of the cluster II (Daims *et al.*, 2015; van Kessel *et al.*, 2015). The genes involved in ammonia oxidization in comammox bacteria are assumed to have been horizontally transferred from β-AOB (Palomo *et al.*, 2018), which presumably affected the conservation of amino acid residues at the HAO active sites in β-AOB and comammox bacteria. HAO active site residues differ between β-AOB and γ-AOB (Fig. 4A), resulting in different HAO inhibitor effects, with phenylhydrazine inducing a 40% reduction in HAO activity in β-AOB, but having no effect on that in γ-AOB (Nishigaya *et al.*, 2016). Nevertheless, the present study revealed the conservation of HAO enzyme active site residues across bacterial taxa in agricultural field soils, suggesting that an HAO inhibitor, such as phenylhydrazine, is capable of suppressing HAO activity in broad nitrifying bacteria inhabiting agricultural fields, including β-AOB and comammox bacteria.

The AOB community structure is affected by various environmental factors, such as soil pH, ammonia affinity, urease activity, and salt tolerance (Koops and Pommerening-Röser, 2001; Tago *et al.*, 2015; Li *et al.*, 2018; Aigle *et al.*, 2019; Hayatsu *et al.*, 2021). Organic fertilizer-treated soils contained higher comammox-*amoA* and *nxrB* copy numbers than chemical fertilizer-treated soils (Table 2). Some *Nitrospira* hydrolyze urea and/or harbor genes that regulate the assimilation of simple organic substrates, such as pyruvate, formate, and acetate (Lucker *et al.*, 2010; Koch *et al.*, 2015), which could be advantageous in an abundance of *Nitrospira* in the organic fertilizer-treated soils.

The comammox-*amoA* gene copy number was markedly higher than the *nxrB* gene copy number in all soil samples, except for those from the chemical fertilizer-treated cabbage field (Table 2), which may be affected by multiple copies of the *amoCAB* gene cluster in comammox genomes (Camejo *et al.*, 2017) and/or differences in PCR amplification efficiency between the two primer sets. The abundances of AOA-*amoA* and AOB-*amoA* were markedly less in chemical fertilizer-treated tea field soil than in all other soils. The abundances of AOB and AOA were previously shown to be markedly affected by soil pH (Li *et al.*, 2018; Aigle *et al.*, 2019). The decreases observed in their abundances in the present study may have been due to the extremely acidic soil (pH: 3.35) of the chemical fertilizer-treated tea field. However, organic fertilizer-treated tea field soil (pH: 3.83) had similar AOA-*amoA* and AOB-*amoA* gene copy numbers to those of AOA-*amoA* and AOB-*amoA* in the other soil samples (Table 2). The relationship between AOA and AOB abundances and nitrogen fertilizer treatment is currently unclear. However, the abundance of AOA may have increased due to organic nitrogen mineralization (Levičnik-Höfferle *et al.*, 2012), and AOB became more abundant at higher soil pH and with the release of ammonia during the mineralization of organic fertilizer (Dai *et al.*, 2021), which could affect increases in AOA and AOB abundances in organic fertilizer-treated tea field soil.

Since AOA do not have canonical *hao* genes, nitrification inhibitors against HAO do not regulate AOA growth or N_2_O emissions from AOA. Moreover, the abundance of AOA-*amoA* ranged from 9.7×10^6^ to 3.9×10^7^ gene copies (g dry soil)^–1^, except for the tea field treated with chemical fertilizer (Table 2). However, AOB, not AOA, are responsible for N_2_O emissions in agricultural soils (Prosser *et al.*, 2020), which indicates that nitrification inhibitors against HAO reduce major N_2_O emissions from agricultural soils.

The *hao* gene diversity of β-AOB was similar in three different fields treated with chemical fertilizer, in which *Nitrosospira* cluster 3b -*N. tenuis*- was dominant. *Nitrosospira* cluster 3b has high ammonia tolerance (Webster *et al.*, 2005). The bacterial growth of *N. tenuis* is slower than that of other AOB, with an optimal temperature and pH of 25–30°C and pH 7.7–7.8, respectively. *N. tenuis* was originally named *Nitrosovibrio tenuis* because its cell morphology is curved rods (Harms *et al.*, 1976). However, the physiological and genomic features of *Nitrosospira* cluster 3b -*N. tenuis*- remain largely unknown because there are few isolates in the cluster, except for the type strain *N. tenuis* Nv1. Further studies are needed to reveal the mechanisms by which *Nitrosospira* cluster 3b -*N. tenuis*- adapts to chemical fertilizer-treated soils. On the other hand, cluster 3a -*Nitrosospira* sp. nsp2- had higher relative abundance in the organic fertilizer-treated soybean field soil, but not in the cabbage and tea fields treated with organic fertilizer. Soil tillage practices affect the community structure of AOB (Haiming *et al.*, 2022). The organic fertilizer-treated soybean field has been maintained under no-tillage management, which might contribute to the unique AOB community that formed in this soil.

An ana­lysis of comammox-*hao* gene diversity revealed that comammox clade B was absent in tea field soils, but dominant in cabbage and soybean field soils (Fig. 2B). Comammox clade A1 was dominant in tea field soils (Fig.‍ ‍2B), which was consistent with previous findings (Takahashi *et al.*, 2020). Since the genomes of comammox clades A and B show high dissimilarity (Palomo *et al.*, 2018), the absence of comammox clade B in tea field soils indicates ecological niche differentiation. Numerous PCR primer sets have been designed for comammox *amoA* (Pjevac *et al.*, 2017; Fowler *et al.*, 2018; Xia *et al.*, 2018; Zhao *et al.*, 2019; Jiang *et al.*, 2020; Lin *et al.*, 2020). However, there is no primer set that amplifies a wide range of *amoA* in the two clades. Therefore, the primer set designed in the present study may not have adequately amplified comammox clade B in the soils tested. The *nxrB* gene amplicon sequence ana­lysis revealed that *Nitrospira* clusters I and V were present in cabbage and soybean field soils, but were absent in tea field soils (Fig. 7). Furthermore, within *Nitrospira* cluster II, the dominant OTUs in tea field soils markedly differed from those in cabbage and soybean field soils, suggesting that the dominant NOB in tea field soils adapted to the extremely low pH. However, the mechanisms underlying this adaptation warrant further investigation.

The present study revealed similar predominant bacterial clusters in ana­lyses of *hao* and *amoA* gene diversities, suggesting that the *hao* gene is also applicable to a phylogenetic ana­lysis of ammonia-oxidizing microorganisms. However, fewer *hao* gene sequences have been deposited in public databases than *amoA* gene sequences. For example, no *hao* gene sequences were available for *Nitrosospira* cluster 2 or 4. AOB diversity has so far been examined by *amoA* and 16S rRNA genes (Purkhold *et al.*, 2000). To fill the gap in data available for *amoA* and *hao* genes, further studies are‍ ‍needed to 1) identify the *hao* gene by the genome sequencing of bacterial isolates and 2) examine *hao* gene diversity in various environments.

## Citation

Ohbayashi, T., Wang, Y., Aoyagi, L. N., Hara, S., Tago, K., and Hayatsu, M. (2023) Diversity of the Hydroxylamine Oxidoreductase (HAO) Gene and Its Enzyme Active Site in Agricultural Field Soils. *Microbes Environ ***38**: ME23068.

https://doi.org/10.1264/jsme2.ME23068

## Supplementary Material

Supplementary Material

## Figures and Tables

**Table 1.** Summary of soil properties and ammonia oxidation potential in this study

Field type	Fertilizer	pH	total C (%)	total N (%)	available P (μg [g dry soil]^–1^)	NH_4_-N (μg N [g dry soil]^–1^)	NO_3_-N (μg N [g dry soil]^–1^)	AOP^a^ (nmole h^–1^ g^–1^)	water content (%)
Cabbage field	Chemical	5.23±0.10	3.11±0.10	0.32±0.02	51.81±1.47	13.84±2.58	54.78±3.07	18.17±1.69	11.77±0.58
	Organic	6.95±0.02	5.16±0.34	0.63±0.03	47.96±2.98	12.92±1.62	206.89±28.08	32.88±4.81	12.00±0.11
Soybean field	Chemical	6.27±0.06	5.00±0.14	0.38±0.01	17.28±2.04	56.55±9.08	36.32±1.35	24.36±3.90	28.34±0.42
	Organic	6.19±0.20	17.79±0.69	1.10±0.04	18.29±3.57	86.98±34.28	343.23±49.82	142.24±7.96	44.00±1.62
Tea field	Chemical	3.35±0.10	31.53±1.76	2.43±0.14	201.40±30.41	37.66±19.90^b^	255.27±34.95	13.81±2.86	62.18±1.67
	Organic	3.83±0.14	18.80±3.43	1.55±0.32	514.42±113.28	124.62±86.84^b^	501.45±112.57	60.03±15.02	53.54±3.50

^a^ AOP: Ammonia oxidation potential ^b^ The values for the mean±SD are of two replicates because one replicate was not detectable.

**Table 2.** Abundances of *amoA* genes of AOA, AOB, and comammox and the *nxrB* gene of NOB in agricultural field soils

Sample Name (gene copy [g dry soil]^–1^)		AOA*-amoA*	AOB*-amoA*	comammox-*amoA*	*nxrB*
Cabbage field	Chemical	1.4×10^7^±1.1×10^6^	3.3×10^6^±5.5×10^5^	9.1×10^5^±2.6×10^5^	5.6×10^6^±1.1×10^6^
	Organic	1.2×10^7^±6.9×10^6^	3.7×10^5^±1.6×10^5^	2.7×10^7^±1.3×10^7^	1.6×10^7^±5.0×10^6^
Soybean field	Chemical	9.7×10^6^±1.8×10^6^	4.6×10^6^±3.3×10^5^	1.5×10^7^±2.9×10^6^	8.2×10^6^±1.5×10^6^
	Organic	2.1×10^7^±6.6×10^6^	4.9×10^6^±4.4×10^5^	5.9×10^7^±6.7×10^6^	2.7×10^7^±3.4×10^5^
Tea field	Chemical	1.7×10^4^±1.1×10^3^	1.8×10^5^±8.9×10^4^	5.3×10^7^±6.1×10^4^	3.9×10^6^±1.7×10^6^
	Organic	3.9×10^7^±4.1×10^6^	5.4×10^6^±3.8×10^6^	1.3×10^8^±8.0×10^5^	8.8×10^6^±6.2×10^6^
