# Supplementary material for: Diversity of the Hydroxylamine Oxidoreductase (HAO) Gene and Its Enzyme Active Site in Agricultural Field Soils
Source: Microbes Environ. 2023 Dec 13;38(4):ME23068. doi: 10.1264/jsme2.ME23068 (PMC10728637; doi:10.1264/jsme2.ME23068)
Supplement: Supplementary file 1 — Supplementary Material [file 38_23068_s1.pdf]

Fig. S1

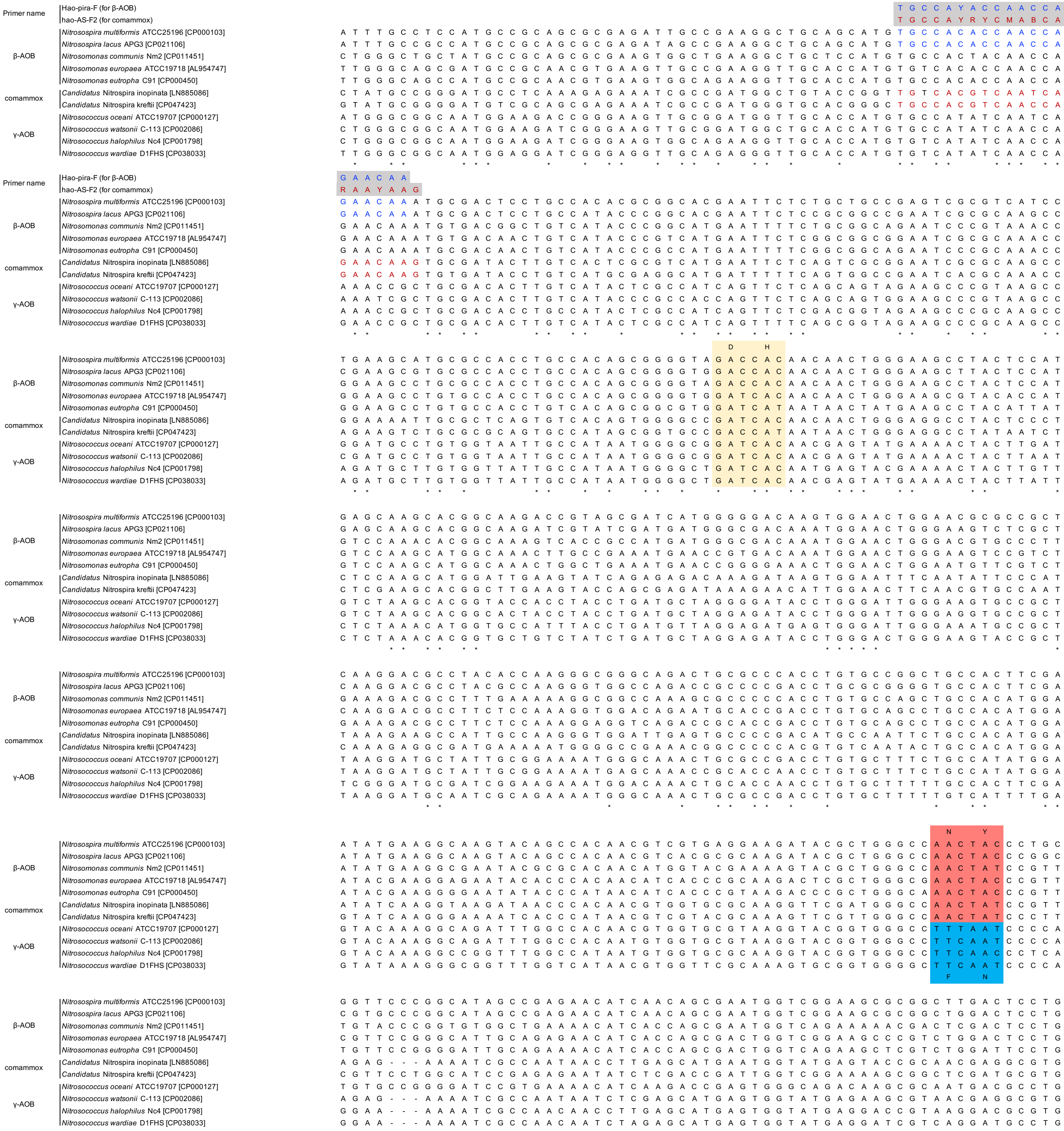

Primer name
Hao-pira-F (for β-AOB)
Hao-AS-F2 (for comammox)
hao-AS-F2 (for comammox)
β-AOB
Nitrosospira multiformis ATCC25196 [CP000103]
Nitrosospira lacus APG3 [CP021106]
Nitrosomonas communis Nm2 [CP011451]
Nitrosomonas europaea ATCC19718 [AL954747]
Nitrosomonas eutropha C91 [CP000450]
comammox
Candidatus Nitrospira inopinata [LN885086]
Candidatus Nitrospira kreftii [CP047423]
γ-AOB
Nitrosococcus oceani ATCC19707 [CP000127]
Nitrosococcus watsonii C-113 [CP002086]
Nitrosococcus halophilus Nc4 [CP001798]
Nitrosococcus wardiae D1FHS [CP038033]

Fig. S1-continued

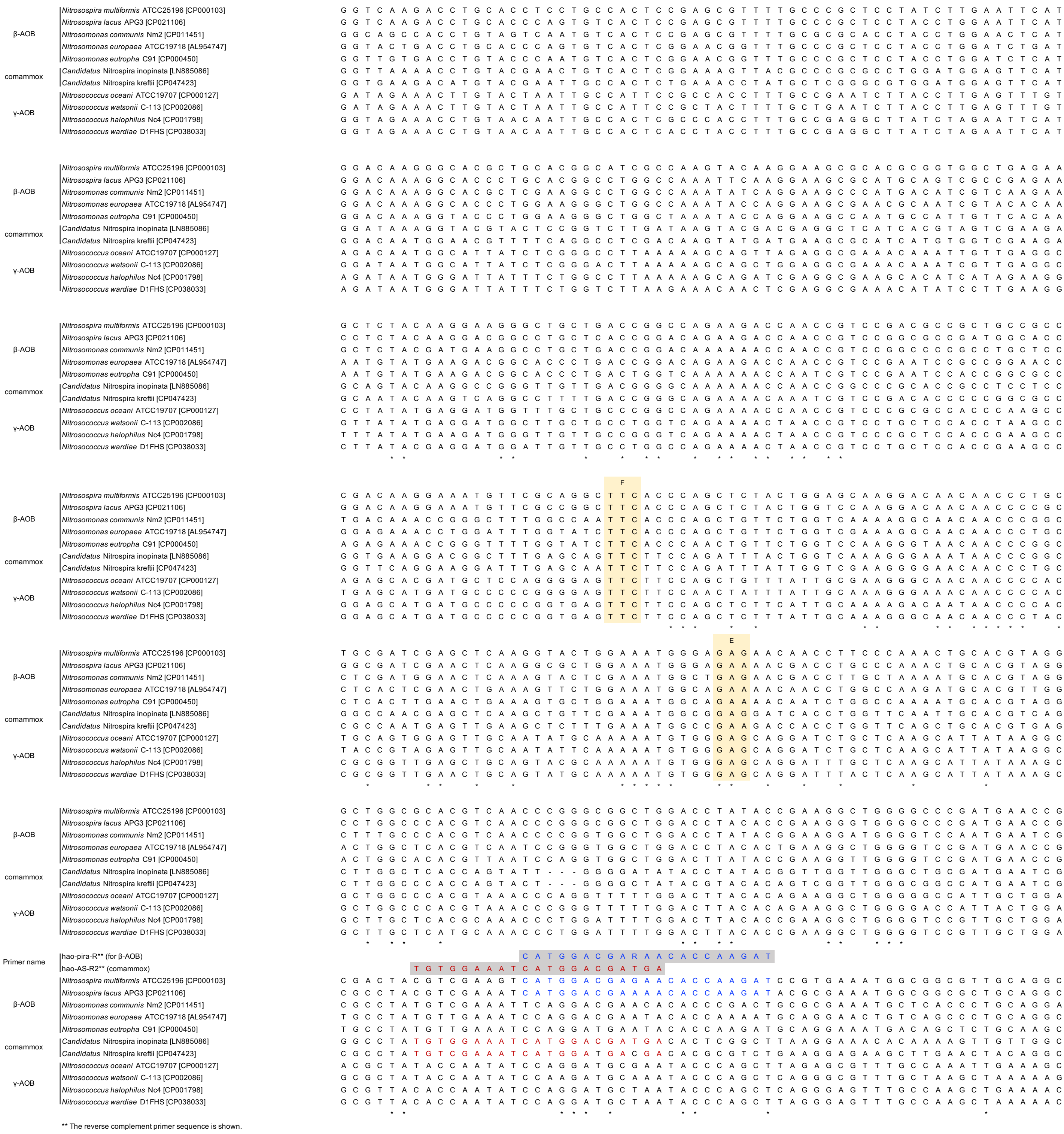

Fig. S1. The primer design for hydroxylamine oxidoreductase (HAO) gene in β-AOB and comammox bacteria. Multiple alignment of HAO gene in β-AOB, comammox and γ-AOB is shown. The colors legends are same as those in Fig. 4. Primer sets for β-AOB-HAO (blue) and for comammox-HAO (red).

Fig. S2

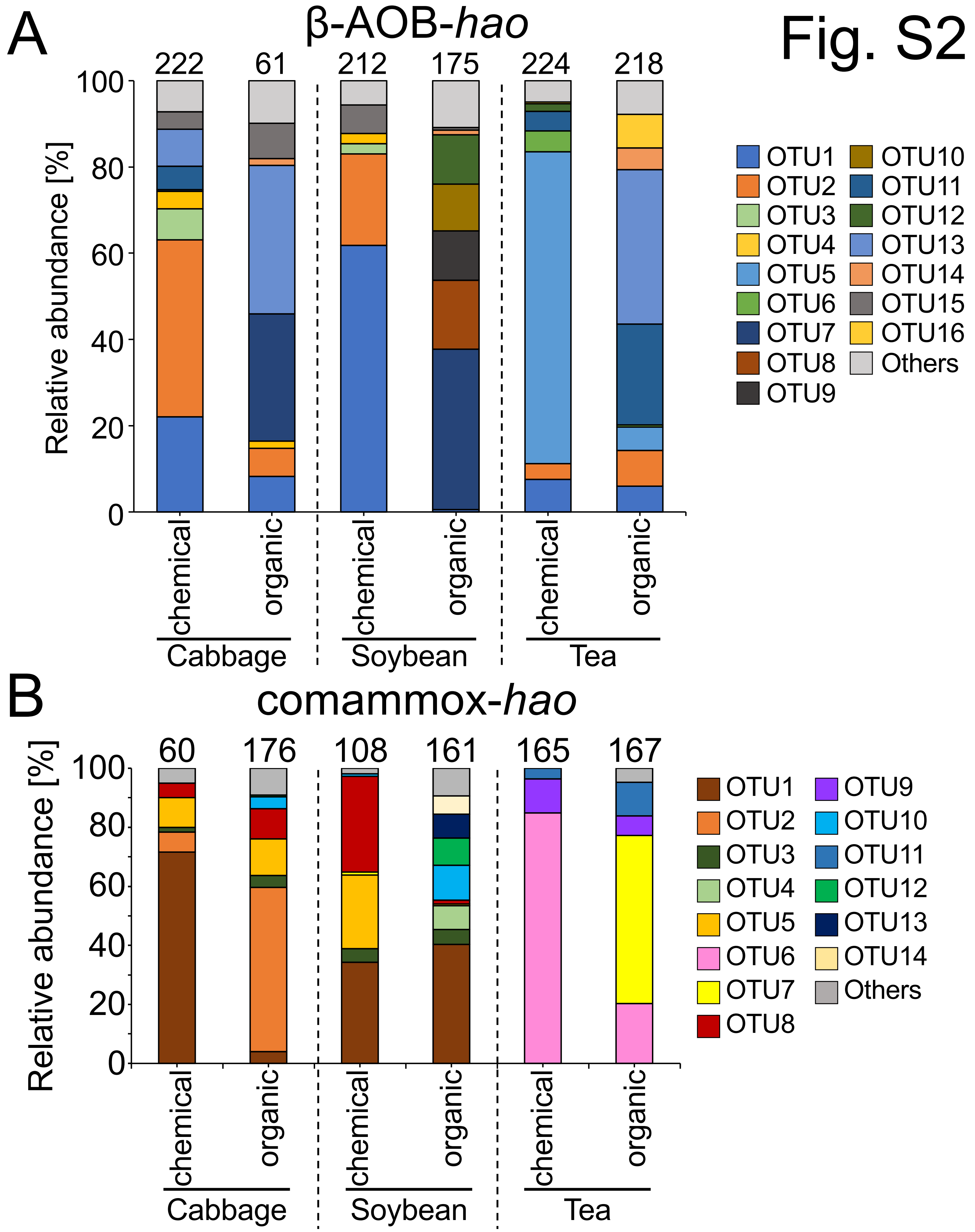

Fig. S2. The *hao* gene diversity of (A) β-AOB and in (B) comammox bacteria in agricultural field soils at OTU level. Total numbers of clones per soil type are shown on the graph. Details are provided in Table S4 and Table S5.

Fig. S3

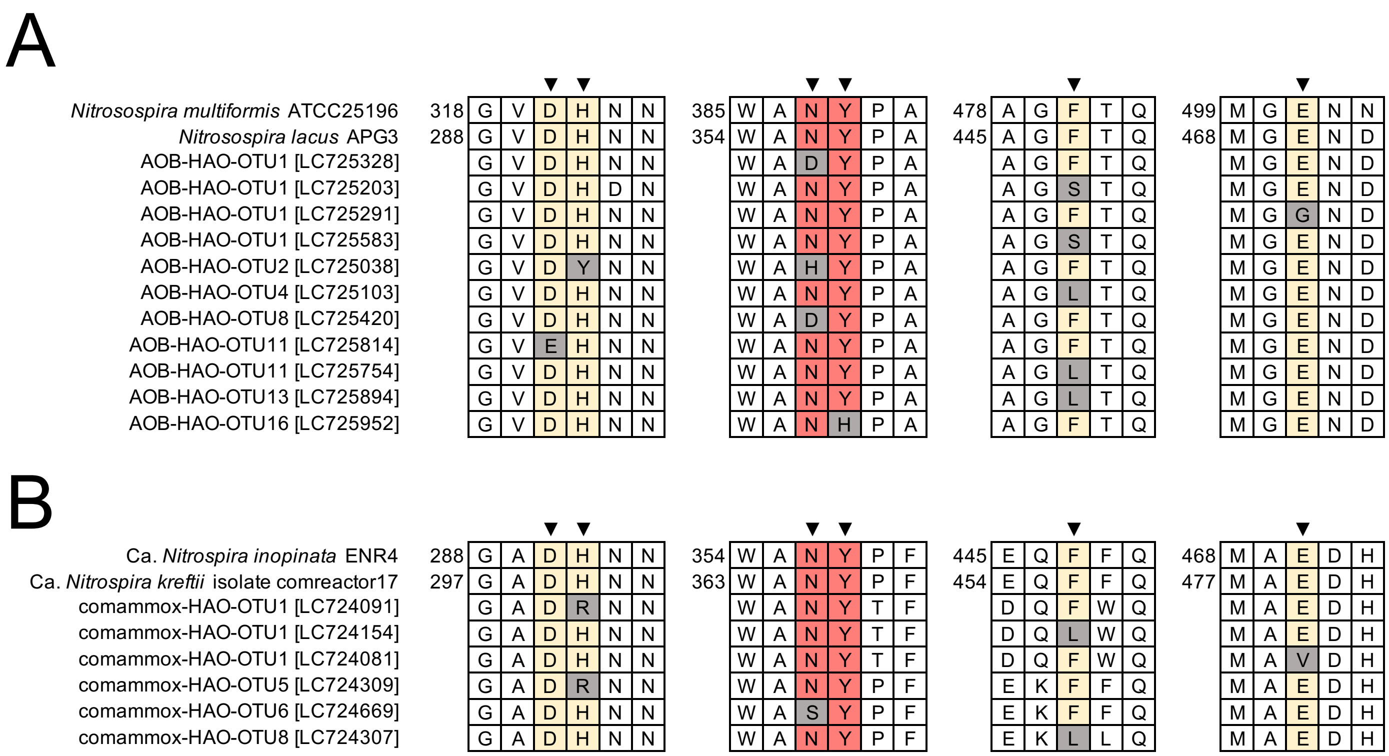

Fig. S3 Different active site residues of hydroxylamine oxidoreductase (HAO) enzyme in (A) β-AOB-*hao* OTUs and (B) comammox-*hao* OTUs. The colors and arrowheads legends are same as those in Fig. 4, and different active site residues (Gray).

Fig. S4

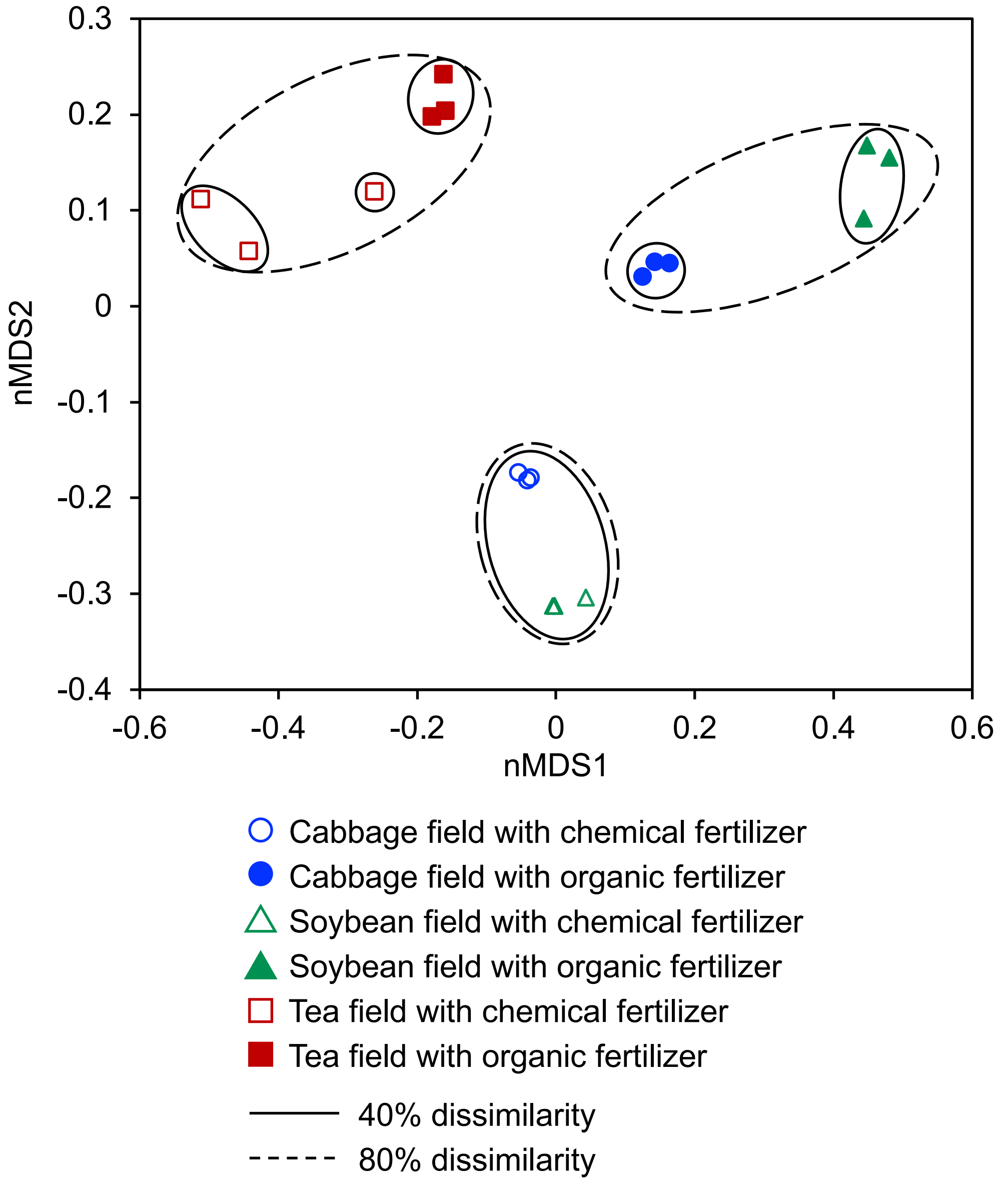

Fig. S4. The nMDS analysis of AOB-*amoA* gene diversity in agricultural field soils. Symbols and colors legends are same as those in Fig. 1. A closed and dot lines indicate 40% and 80% dissimilarities, respectively.

Fig. S5

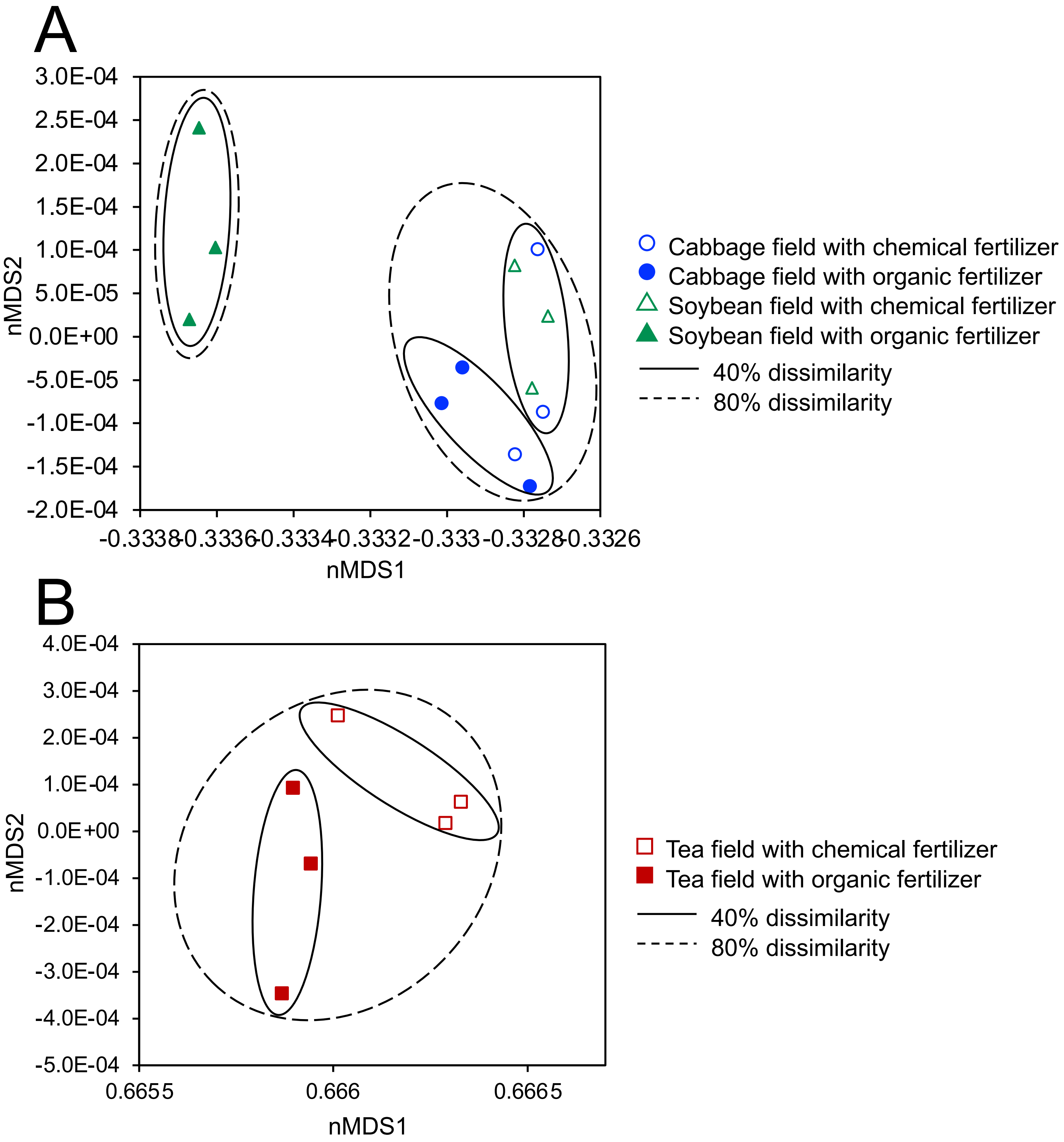

Fig. S5. The nMDS analysis of comammox-*amoA* gene diversity in (A) cabbage and soybean field soils and in (B) tea field soil. Symbols and those colors are as shown in Fig. 1. Closed and dot lines indicate 40% and 80% dissimilarities, respectively.

Fig. S6

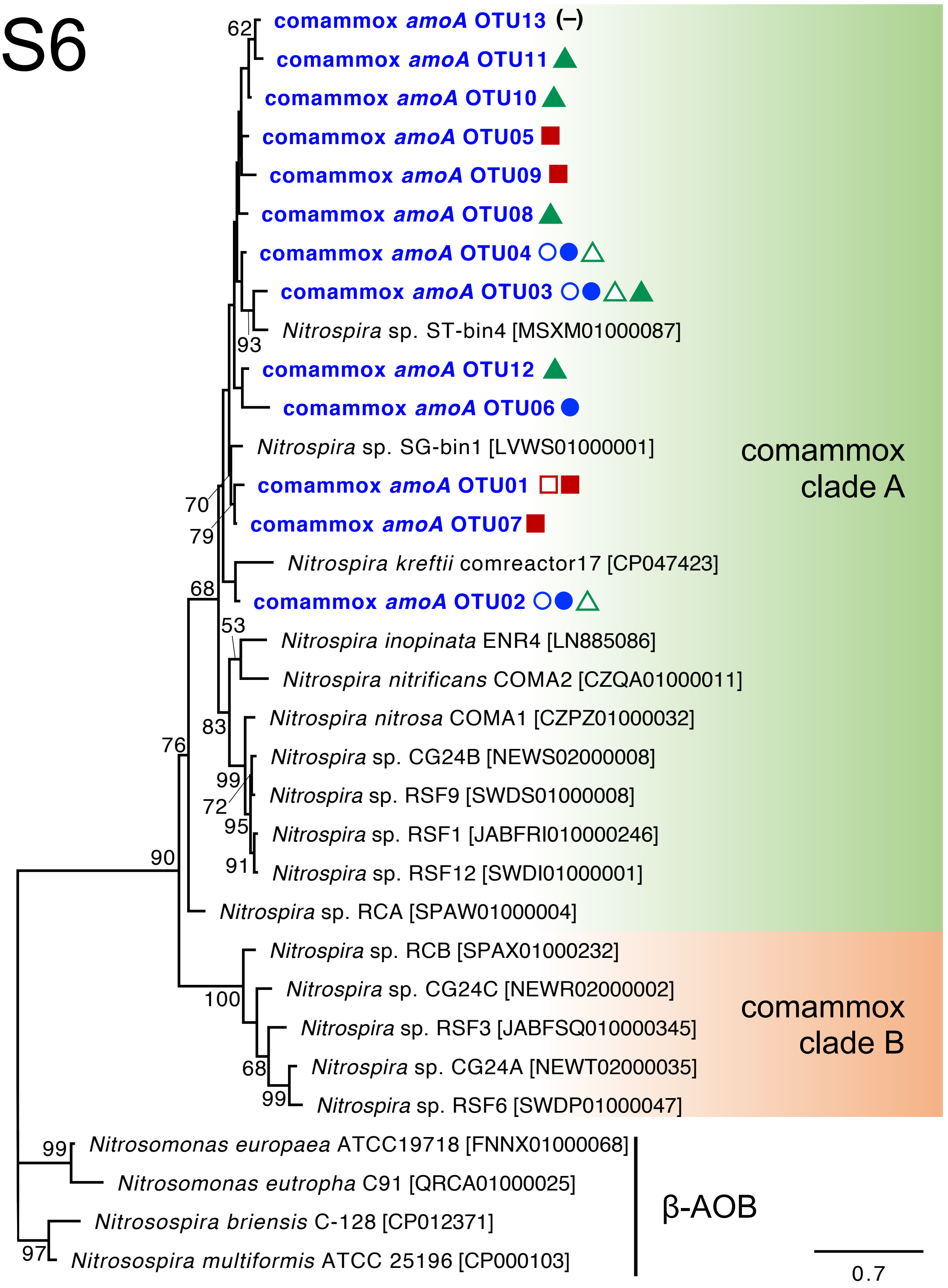

Fig. S6. Molecular phylogenetic analysis of comammox-*amoA* gene diversity in agricultural field soils. The ML tree was generated based on 203 aligned nucleotide sites of the *amoA* gene. The ML bootstrap values (%) was calculated with 1,000 replicates, and bootstrap values > 50 are shown at tree nodes. Accession numbers in the DNA databases (DDBJ/EMBL/GenBank) are shown in square brackets. We referred to the nucleotide sequence information of comammox bacteria and its criteria reported in a previous study (Palomo *et al*., 2022). Representative comammox-*amoA* OTUs are shown in blue color with bold case. Symbols and colors legends are same as those in Fig. 1.

Fig. S7

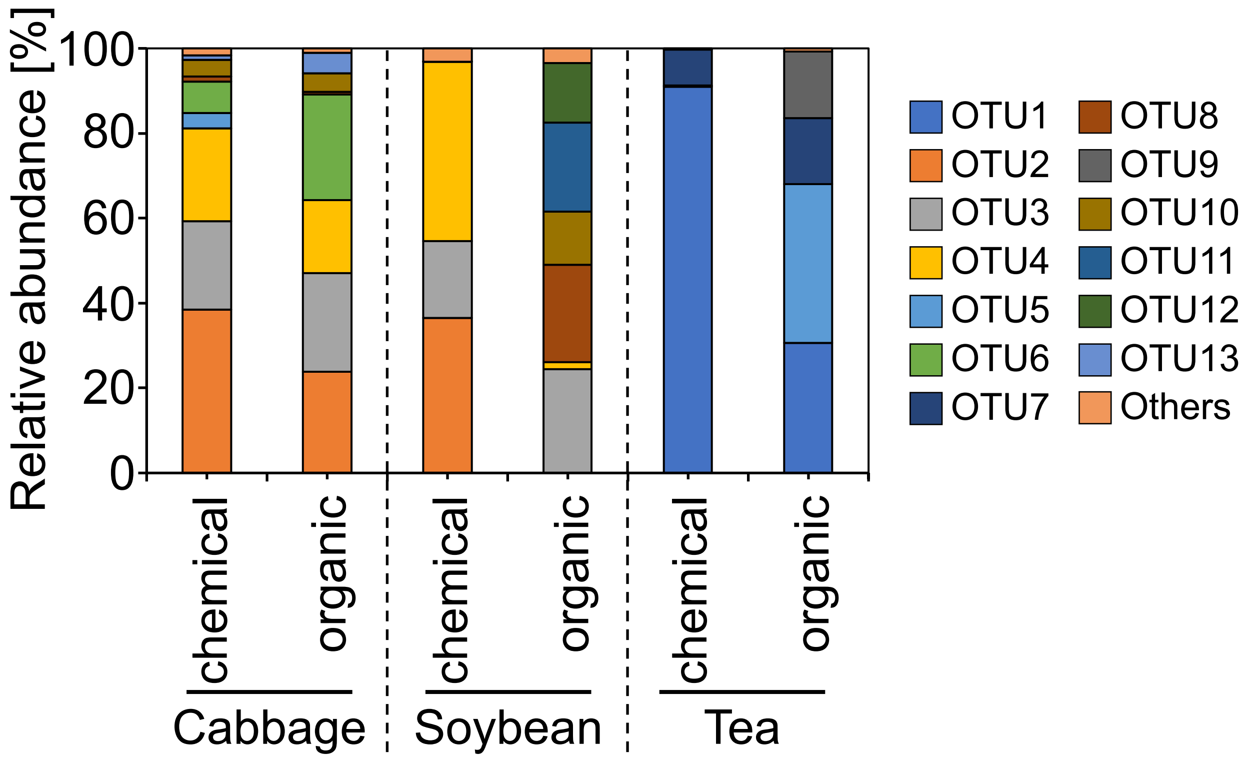

Fig. S7. The comammox-*amoA* gene diversity in Japanese agricultural fields at OTU level. Details of number of sequence reads are provided in Table S7

Fig. S8

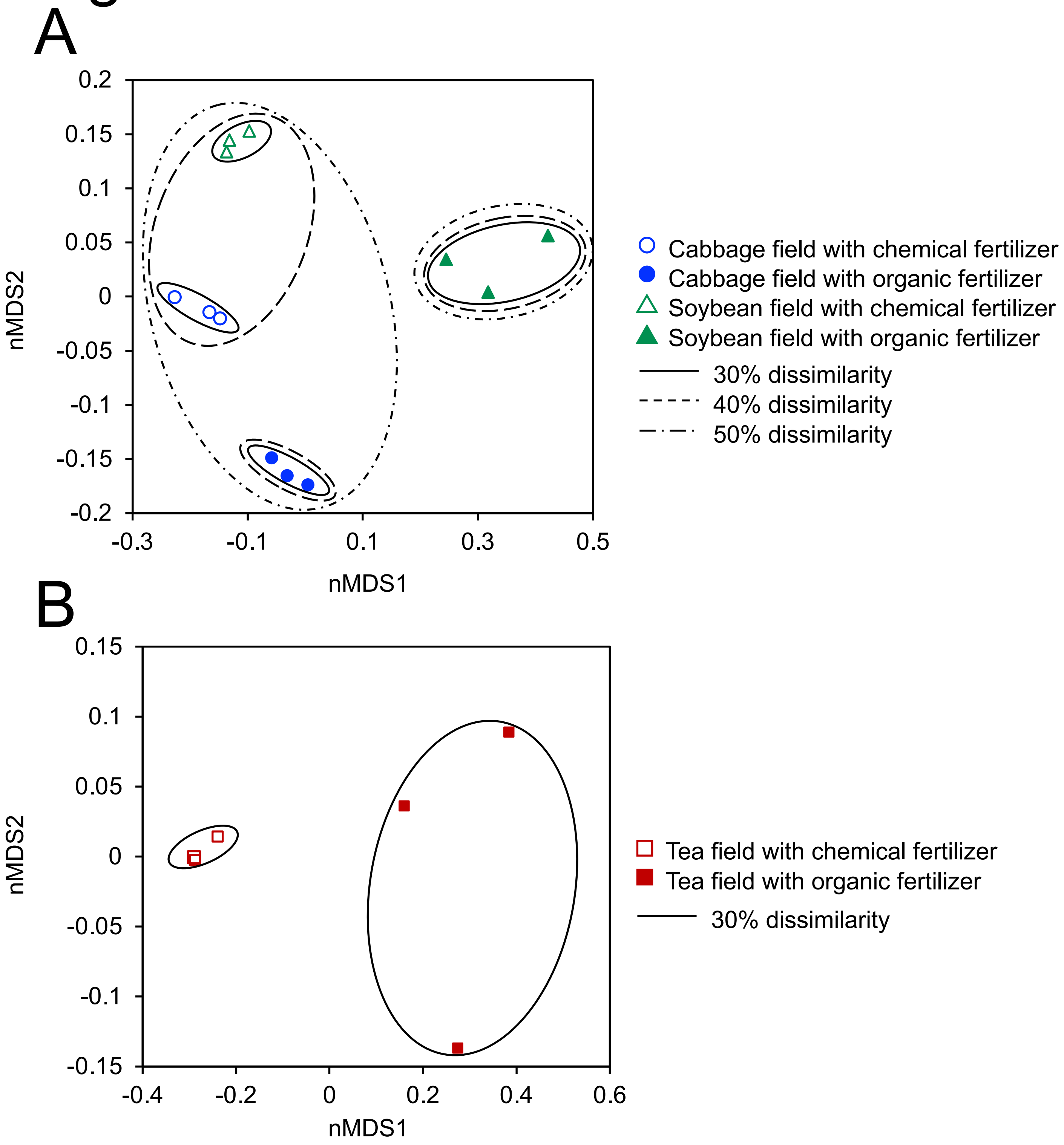

Fig. S8. The nMDS analysis of *nxrB* gene diversity in (A) cabbage and soybean fields and in (B) tea field in Japan. Symbols and those colors are as shown in Fig. 1. Closed, dashed and dashed-and-dotted lines indicate 30%, 40% and 50% dissimilarities, respectively.

Fig. S9

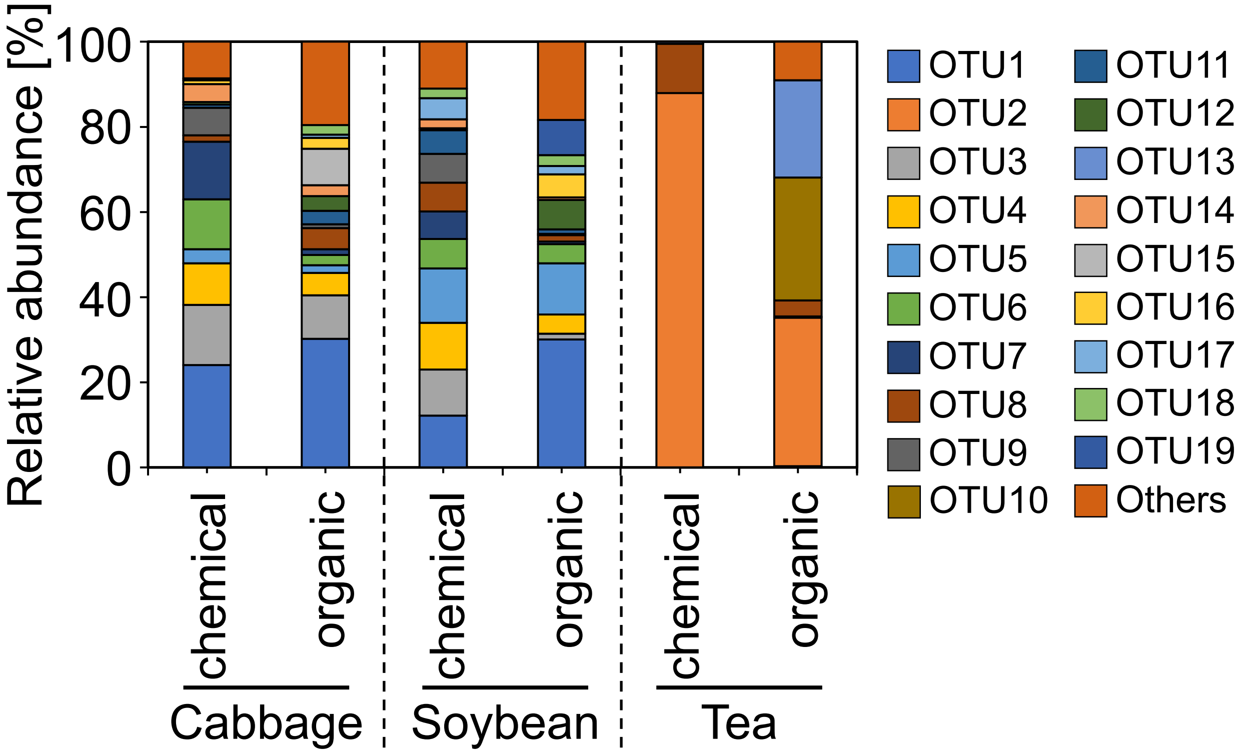

Fig. S9. The *nxrB* gene diversity in agricultural fields at OTU level. Details of sequence reads are provided in Table S8.

Fig. S10

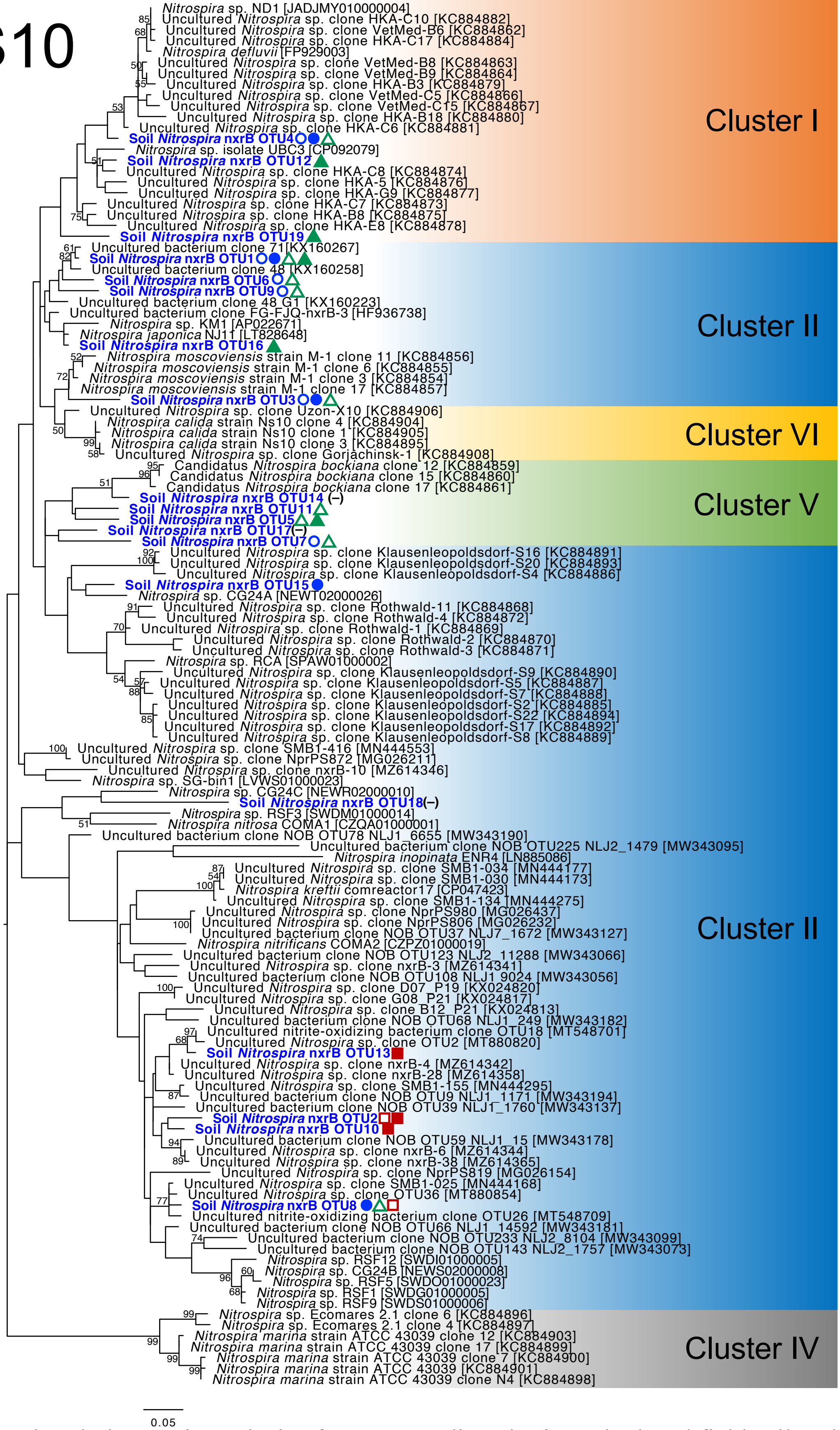

Fig. S10. Molecular phylogenetic analysis of *nxrB* gene diversity in agricultural field soils. The ML tree was generated based on 229 aligned nucleotide sites of the *nxrB* gene. The ML bootstrap values (%) was calculated with 1,000 replicates. Bootstrap values > 50 are shown at tree nodes. Accession numbers deposited in DNA databases (DDBJ/EMBL/GenBank) are shown in square brackets. Representative *nxrB* OTUs are shown in blue color with bold case. Symbols and colors legends are same as those in Fig. 1.

Table S1. Soil samples used for cloning and sequencing in this study

Field type	Fertilizer type	Fertilizer amount (kgN / ha)	Accession No.				
			AOB-*hao*	comammox-*hao*	AOB-*amoA*	comammox-*amoA*	*nxrB*
Cabbage field	Chemical	250	LC724905–LC725126	LC724068–LC724127	DRR400628–DRR400630	DRR400646–DRR400648	DRR400664–DRR400666
	Organic	750*	LC725127–LC725187	LC724128–LC724303	DRR400631–DRR400633	DRR400649-DRR400651	DRR400667–DRR400669
Soybean field	Chemical	300	LC725188–LC725399	LC724304–LC724411	DRR400634–DRR400636	DRR400652–DRR400654	DRR400670–DRR400672
	Organic	ND**	LC725400–LC725574	LC724412–LC724572	DRR400637–DRR400639	DRR400655–DRR400657	DRR400673–DRR400675
Tea field	Chemical	312 (496***)	LC725575–LC725798	LC724573–LC724737	DRR400640–DRR400642	DRR400658–DRR400660	DRR400676–DRR400678
	Organic	520	LC725799–LC726016	LC724738–LC724904	DRR400643–DRR400645	DRR400661–DRR400663	DRR400679–DRR400681

*Cow manure compost was applied.

**7000 kgC of leaf litter compost was applied per hectare.

***Since chemical fertilizer alone is not suitable for the growth of tea plants, 31.2 kg of chemical nitrogen fertilizer and 18.4 kg of organic nitrogen fertilizer were applied.

Table S2. Primer sets for qPCR, clone library analysis and amplicon sequence.

Target gene	Primer name	sequence (5' to 3')	Annealing temperature (ºC)	References
**For qPCR**				
AOA-*amoA*	*amoA* -19IF	ATGGTCTGGCTIAGACG	56	Morimoto *et al*., 2011
	amoA -616R	GCCATCCATCTGTATGTCCA		Tourna *et al*., 2008; Yang *et al*., 2017
AOB-*amoA*	*amoA* -1F	GGGGTTTCTACTGGTGGT	54	Rotthauwe *et al*., 1997; Yang *et al*., 2017
	amoA -2R-GG	CCCCTCGGGAAAGCCTTCTTC		Nicolaisen and Ramsing, 2002
comammox-*amoA*	comaA-*amoA* -F	CBKCNTGGTGGTGGTGGTC	65	This study
	comaA-*amoA* -R	AGCCCATRTAGTCNGCCC		
nxrB	*nxrB* -F	GTGGTGGAACAAYGTSGARAC	60	This study
	nxrB -R1	GCATCGABGTNGSVGTRTC		
**For clone library analysis**				
β-AOB-*hao*	*hao* -pira-F	TGCCAYACCAACCAGAACAA	50	This study
	hao -pira-R	ATCTTGGTGTTYTCGTCCATG		
comammox-*hao*	*hao* -AS-F2	TGCCAYRYCMABCARAAYAAC	55	This study
	hao -AS-R2	TCMTCRTCCATGATTTCSACA		
**For amplicon sequence**				
AOB-*amoA*	*amoA* -1F	GGGGTTTCTACTGGTGGT	54	Rotthauwe *et al*., 1997; Yang *et al*., 2017
	amoA -2R	CCCCTCGGGAAAGCCTTCTTC		Nicolaisen and Ramsing, 2002
comammox-*amoA*	comaA-*amoA* -F	CBKCNTGGTGGTGGTGGTC	65	This study
	comaA-*amoA* -R	AGCCCATRTAGTCNGCCC		
nxrB	*nxrB* -F	GTGGTGGAACAAYGTSGARAC	60	This study
	nxrB -R1	GCATCGABGTNGSVGTRTC		

Table S3. Correlation between the bacterial gene abundance detected by qPCR and environmental factors

Environmental factors	AOA-*amoA* ρ	AOB-*amoA* ρ	comammox-*amoA* ρ	*nxrB* ρ
pH	0.55 *	0.088	-0.28	0.62 **
total C	-0.39	-0.038	0.86 ***	0.044
total N	-0.52 *	-0.15	0.84 ***	-0.030
available P	-0.58 *	-0.27	0.35	-0.46
NH_4-N	0.15	0.39	0.17	0.12
NO_3-N	-0.15	0.22	0.81 ***	0.26
AOP	0.44	0.69 **	0.43	0.78 ***
water content	-0.52 *	0.015	0.74 ***	-0.16

ρ, Spearman's rho (Spearman's rank correlation coefficient).

* p<0.05; ** p<0.005; ***p<0.005

Table S4. OTU profile of the β-AOB-*hao* gene diversity in agricultural fields

Cluster name	OTU No.	Cabbage field		Soybean field		Tea field		total clone
		Chemical	Organic	Chemical	Organic	Chemical	Organic	number
Cluster 3b -*N. tenuis*-	OTU1	49	5	131	1	17	13	216
	OTU2	91	4	45	0	8	18	166
	OTU3	16	0	5	0	0	0	21
	OTU4	9	1	5	0	0	0	15
	OTU5	0	0	0	0	162	12	174
	OTU6	0	0	0	0	11	1	12
Cluster 3a -*Nitrosospia* sp. nsp2-	OTU7	1	18	0	65	0	0	84
	OTU8	0	0	0	28	0	0	28
	OTU9	0	0	0	20	0	0	20
	OTU10	0	0	0	19	0	0	19
Nitrosospira sp. 56-18 cluster	OTU11	12	0	0	0	10	51	73
	OTU12	0	0	0	20	4	0	24
Cluster 3a -*N. multiformis* -	OTU13	19	21	0	0	0	78	118
Cluster 3b -*N. briensis* -	OTU14	0	1	0	2	0	11	14
Cluster 0	OTU15	9	5	14	1	0	0	29
	OTU16	0	0	0	0	1	17	18
Others	Others	16	6	12	19	11	17	81
	total	222	61	212	175	224	218	1112

Table S5. OTU profile of the comammox-*hao* gene diversity in agricultural fields

Cluster name	OTU No.	Cabbage field		Soybean field		Tea field		total clone number
		Chemical	Organic	Chemical	Organic	Chemical	Organic	
Clade B	OTU1	43	7	37	65	0	0	152
	OTU2	4	98	0	0	0	0	102
Clade A2	OTU3	1	7	5	8	0	0	21
	OTU4	0	0	0	13	0	0	13
Clade A1	OTU5	6	22	27	1	0	0	56
	OTU6	0	0	0	0	140	34	174
	OTU7	0	0	1	0	0	95	96
	OTU8	3	18	35	2	0	0	58
	OTU9	0	0	0	0	19	11	30
	OTU10	0	7	1	19	0	0	27
	OTU11	0	0	0	0	6	19	25
	OTU12	0	1	0	15	0	0	16
	OTU13	0	0	0	13	0	0	13
	OTU14	0	0	0	10	0	0	10
Others	Others	3	16	2	15	0	8	44
total	total	60	176	108	161	165	167	837

Table S6. OTU profile of the AOB-*amoA* gene diversity in agricultural field soils

		Cabbage field						Soybean field						Tea field					
Cluster name	OTU No.	Chemical #1	Chemical #2	Chemical #3	Organic #1	Organic #2	Organic #3	Chemical #1	Chemical #2	Chemical #3	Organic #1	Organic #2	Organic #3	Chemical #1	Chemical #2	Chemical #3	Organic #1	Organic #2	Organic #3
Cluster 3b -*N. tenuis* -	OTU1	33	1	0	187	9	0	0	0	0	0	0	0	35869	38612	18499	2633	7274	2056
Cluster 3a -*N. multiformis* -	OTU2	9356	7153	7159	8500	12883	11240	57	29	42	51	0	0	9	13	1055	7735	8054	6913
Cluster 3a -*Nitrosospira* sp. nsp2-	OTU3	30	93	89	13492	11134	11533	0	0	24	12933	15054	12370	0	0	0	0	0	0
Cluster 3b -*N. tenuis* -	OTU4	5824	5537	5717	1988	1093	2964	22463	22330	20066	61	48	24	0	0	0	0	0	0
Cluster 3b -*N. tenuis* -	OTU5	8176	7532	7923	255	97	571	3775	3852	3746	0	0	0	2151	690	6516	3559	4456	2178
Nitrosospira sp. 56-18 cluster	OTU6	3226	5543	3225	1693	3146	4082	190	94	125	5	11	16	1295	355	2410	12715	3607	3146
Cluster 4	OTU7	3772	2574	3330	6998	8770	4896	5354	5503	7235	1133	947	360	0	0	0	0	0	0
Cluster 2	OTU8	0	0	0	0	185	101	0	32	0	0	0	0	873	489	5681	8229	1174	21955
Cluster 3b -*N. briensis* -	OTU9	28	32	11	216	0	2154	0	0	0	494	3717	148	0	0	0	2231	9248	2864
Cluster 3a -*Nitrosospira* sp. nsp2-	OTU10	0	0	0	90	0	0	37	0	0	12251	9874	14305	0	0	0	0	0	0
Cluster 3b -*N.tenuis* -	OTU11	3726	6957	8785	87	39	87	3359	3598	2401	153	0	0	0	0	0	13	0	0
Cluster 4	OTU12	5204	4302	3498	14	0	36	4933	4642	6037	113	70	0	0	0	0	0	0	0
Nitrosospira sp. 56-18 cluster	OTU13	0	0	14	420	51	860	0	0	0	4877	2945	1602	0	0	3271	583	78	7
Cluster 3a -*N. multiformis* -	OTU14	0	0	0	0	0	0	0	0	0	0	0	0	0	0	0	1930	5937	967
Cluster 3b -*Nitrosospira* sp. nsp2-	OTU15	0	0	0	0	0	0	0	0	0	4919	3502	7878	0	0	0	0	0	0
Cluster 0	OTU16	54	11	4	2607	844	647	0	4	450	1276	1509	899	6	0	0	0	0	0
–	Others	995	689	669	3877	2173	1253	256	340	298	2158	2747	2822	221	265	2992	796	596	338
	Total	40424	40424	40424	40424	40424	40424	40424	40424	40424	40424	40424	40424	40424	40424	40424	40424	40424	40424

Table S7. OTU profile of the comammox-*amoA* gene diversity in agricultural field soils

	Cabbage field						Soybean field						Tea field					
Group	Chemical #1	Chemical #2	Chemical #3	Organic #1	Organic #2	Organic #3	Chemical #1	Chemical #2	Chemical #3	Organic #1	Organic #2	Organic #3	Chemical #1	Chemical #2	Chemical #3	Organic #1	Organic #2	Organic #3
OTU1	0	0	0	0	0	0	0	0	0	0	0	0	2826	3290	3151	987	576	1564
OTU2	1110	1157	1657	599	859	967	1451	1350	924	0	0	0	0	0	0	0	0	0
OTU3	645	1154	317	678	656	1037	355	500	987	1159	238	1093	0	0	0	0	0	0
OTU4	759	577	902	440	916	396	1505	1356	1438	82	88	0	0	0	0	0	0	0
OTU5	53	164	154	0	0	0	0	0	0	0	0	0	29	0	0	997	1614	1203
OTU6	463	140	148	1271	675	600	0	0	0	0	0	0	0	0	0	0	0	0
OTU7	0	0	0	0	0	0	0	0	0	0	0	0	508	108	247	1093	119	359
OTU8	0	8	108	0	0	51	0	9	0	593	976	774	0	0	0	0	0	0
OTU9	0	0	0	0	0	0	0	0	0	0	0	0	0	0	0	293	1037	272
OTU10	212	183	0	287	66	100	0	0	0	367	613	288	0	0	0	0	0	0
OTU11	0	0	0	0	0	0	0	0	0	591	888	654	0	0	0	0	0	0
OTU12	0	0	0	0	0	0	0	0	0	498	506	435	0	0	0	0	0	0
OTU13	122	0	0	73	206	211	0	0	0	0	0	0	0	0	0	0	0	0
Others	34	15	112	50	20	36	87	183	49	108	89	154	35	0	0	28	52	0
Total	3398	3398	3398	3398	3398	3398	3398	3398	3398	3398	3398	3398	3398	3398	3398	3398	3398	3398

Table S8. OTU profile of the *nxrB* gene diversity in agricultural field soils

		Cabbage field						Soybean field						Tea field					
Cluster name	OTU No.	Chemical #1	Chemical #2	Chemical #3	Organic #1	Organic #2	Organic #3	Chemical #1	Chemical #2	Chemical #3	Organic #1	Organic #2	Organic #3	Chemical #1	Chemical #2	Chemical #3	Organic #1	Organic #2	Organic #3
Cluster II	OTU1	2042	2119	1656	2340	2207	2772	891	1040	1005	2417	2456	2391	0	0	0	0	67	0
Cluster II	OTU2	0	0	0	0	0	0	0	0	0	0	0	0	6333	7446	7528	2136	2538	3774
Cluster II	OTU3	1316	1134	982	657	888	923	736	939	944	132	220	0	0	0	0	0	0	0
Cluster I	OTU4	749	894	732	574	374	336	839	813	1021	400	321	349	0	0	0	0	0	0
Cluster V	OTU5	250	318	205	142	243	55	994	1085	1010	1218	681	1035	0	0	0	0	0	0
Cluster II	OTU6	863	776	1204	92	205	263	687	565	413	413	387	281	0	0	0	0	0	0
Cluster V	OTU7	958	909	1420	145	184	0	682	400	501	0	134	0	0	0	0	36	0	23
Cluster II	OTU8	116	153	102	332	394	485	613	521	490	63	130	180	1621	626	544	503	0	419
Cluster II	OTU9	517	528	521	83	60	80	638	469	542	0	60	0	0	0	0	0	0	0
Cluster II	OTU10	0	0	0	0	0	0	0	0	0	0	0	0	0	0	0	3427	1982	1588
Cluster V	OTU11	81	95	0	202	258	310	590	553	197	94	193	0	0	0	0	0	0	0
Cluster I	OTU12	97	0	49	376	205	237	41	0	66	581	417	648	0	0	0	0	0	0
Cluster II	OTU13	0	0	0	0	0	0	0	0	0	0	0	0	118	0	0	1140	2333	2072
Cluster V	OTU14	260	420	319	203	277	151	123	287	120	51	101	0	0	0	0	0	0	0
Cluster II	OTU15	0	0	0	590	773	713	0	0	0	0	0	0	0	0	0	0	0	0
Cluster II	OTU16	107	79	62	239	160	200	0	0	0	258	731	324	0	0	0	0	0	0
Cluster V	OTU17	0	0	0	41	84	56	492	314	378	209	111	162	0	0	0	0	0	0
Cluster II	OTU18	23	50	27	169	175	221	140	231	195	124	162	315	0	0	0	0	0	0
Cluster I	OTU19	0	0	0	0	0	0	0	0	0	765	686	581	0	0	0	0	0	0
	Others	693	597	793	1887	1585	1270	606	855	1190	1347	1282	1806	0	0	0	830	1152	196
	Total	8072	8072	8072	8072	8072	8072	8072	8072	8072	8072	8072	8072	8072	8072	8072	8072	8072	8072
